# Modeling tuberculosis dynamics with the presence of hyper-susceptible individuals for Ho Chi Minh City from 1996 to 2015

**DOI:** 10.1186/s12879-018-3383-3

**Published:** 2018-10-01

**Authors:** Dao Nguyen Vinh, Dang Thi Minh Ha, Nguyen Thi Hanh, Guy Thwaites, Maciej F. Boni, Hannah E. Clapham, Nguyen Thuy Thuong Thuong

**Affiliations:** 1 Wellcome Trust Major Overseas Programme, Hospital for Tropical Diseases, Oxford University Clinical Research Unit, 764 Vo Van Kiet Street, District 5, Ho Chi Minh City, Vietnam; 2 Pham Ngoc Thach Hospital, Ho Chi Minh City, Vietnam; 3 Centre for Tropical Medicine and Global Health, Nuffield Department of Medicine, University of Oxford, Oxford, UK; 4 Center for Infectious Disease Dynamics, Department of Biology, Pennsylvania State University, Pennsylvania, USA

**Keywords:** Tuberculosis, AIDS, Hyper-susceptible, Maximum likelihood estimation

## Abstract

**Background:**

The depletion of CD4 cell is the underlying reason for TB hyper-susceptibility among people with HIV. Consequently, the trend of TB dynamics is usually hidden by the HIV outbreak.

**Methods:**

Here, we aim to evaluate the trend of TB dynamics quantitatively by a simple mathematical model using the known prevalence of hyper-susceptible individuals in the population. In order to estimate the parameters governing transmission we fit this model in a maximum likelihood framework to both reported TB cases and data from samples tested with Interferon Gamma Assay from Ho Chi Minh City - a city with high TB transmission and strong synchronization between HIV/AIDS and TB dynamics.

**Results:**

Our results show that TB transmission in HCMC has been declining among people without HIV; we estimate a 18% (95% CI: 9–25%) decline in the transmission parameter between 1996 and 2015. Furthermore, we show that co-infected patients have limited contribution to the transmission process. For hyper-susceptible individuals, our model suggests that the risk of a new active TB infection occurring is significantly higher than the risk of relapsed active TB, while this is not the case for people without hyper-susceptibility.

**Conclusions:**

The increase of TB notifications in Ho Chi Minh City from 1996 to 2008 is evitable when, as occurred, the number of hyper-susceptible individuals increased faster than the decrease of TB transmission rate. The sharp decrease in TB notifications observed in this city from 2008 to 2015 is the combined result of the decrease of TB transmission rate and the decrease of hyper-susceptible individuals in the population. For hyper-susceptible individuals, we propose that the reason for the reduced relapsed active TB risk is HIV treatment delay. According to HIV treatment guidelines issued by Vietnam’s Ministry of Health, hyper-susceptible individuals usually have to wait until their CD4 cell count falls under 350 cells/μl to start ART. Once patients begin ART, they will remain on ART for the rest of their life and thus have greater protection against relapses of TB. We therefore hypothesize that the delay in using ART imposes considerable TB burden on HCMC despite the declining transmission process.

**Electronic supplementary material:**

The online version of this article (10.1186/s12879-018-3383-3) contains supplementary material, which is available to authorized users.

## Background

Among infectious diseases, tuberculosis (TB) is a leading cause of mortality worldwide with 1.5 million deaths annually, mainly occuring in low and middle income countries [1]. In Vietnam, TB is still one of the top ten leading causes of death among all ages [2] despite substantial attempts to reduce its burden. The Direct Observed Treatment, Short Course (DOTS), the heart of the TB elimination strategy recommended by the World Health Organization (WHO) [3, 4], was implemented in Vietnam in 1994. By 1998 it covered about 96% of the Vietnamese population [5, 6]. After DOTS was adopted, a large amount of National Tuberculosis Control Program’s (NTP) funding was spent on public engagement to educate people about how to prevent transmission of TB in the population. However, annual TB notifications in Vietnam increased from around 75 per 100,000 in 1996 to over 100 per 100,000 in 1998, and remained stable at about 110 per 100,000 until 2014 [6, 7]. In Ho Chi Minh City (HCMC), the biggest city in Vietnam and a high TB transmission setting, an advanced monitoring program for TB relapses in which individuals with active TB are monitored more carefully was implemented by the NTP in 2003. In this program, patients who receive successful treatment are asked to come back to district tuberculosis units (DTUs) for a TB examination two to four months after treatment. However, the number of TB notifications in HCMC increased from about 10,000 in 1996 to about 13,000 in 2008. Subsequently, the number of notifications declined until 2015 – the last year that the TB data used in this paper are available.

It is important to emphasize that when DOTs was implemented, HIV had started to spread widely in the Vietnamese population. The first HIV case in Vietnam was detected in 1990 in HCMC. Two years later, only 11 HIV cases had been reported [8], and in 1993, the first cases of AIDS in Vietnam were reported. The reported number of new AIDS cases per year has increased sharply since then, peaking in 2008 at around 17,000. After that, like TB, this number also appeared to decline until 2015 – the last year that the HIV/AIDS data used in this paper are available. In HCMC, the Vietnam HIV program estimates the numbers of people with HIV and AIDS in 2015 were about 50,000 and 20,000 respectively [9]. Hence, the total number of people living with HIV in HCMC in 2015 was about 70,000, approximately 0.8% to 1% of the HCMC population.

The emergence of HIV/AIDS to human population enhanced the tuberculosis dynamics by creating a massive number of TB hyper-susceptible individuals in the human population. Consequently, high HIV prevalence settings such as Africa becomes target of many modelling HIV/TB studies such as [10–15]. The studies [11, 15] offers an overview about HIV/TB about nine different African countries. There was a strong synchronization between HIV and TB dynamics in these countries. When the HIV prevalence peaks at 10–25%, the number of TB notifications are about five times higher (at about 800 notifications/year). These studies also shows that ART wide use in the population benefits the TB dynamics. The study [10] only focused on the HIV/TB in a South Afican township indicates that HIV positive may account for 75% of TB notifications in a high HIV prevalence area. In the low HIV prevalence settings (≤ 1% HIV prevalence), HIV positives also accounts for a substantial part of TB patients. From October 2003 to Febuary 2005, 40% of TB patients were diagnosed with HIV positive in a small non-modelling study conducted in Cambodia – a developing country with only 0.5% HIV prevalence [16]. In Thailand – a high TB burden country with low HIV prevalence, strong correlation between HIV/AIDS and TB dynamics was observed for past 20 years [7].

Unlike the HIV/TB modeling works cited in this paper which focuses on the effect of various measures, the main aim of this paper is quantitatively investigating the trend of TB transmission in HCMC from 1996 to 2015 – period that HIV/AIDS emerged to HCMC’s population. In order to archive this aim, we need to identify the impact of HIV/AIDS to TB dynamic in HCMC’s population. Because we use multiple data sources for analyzing HCMC’s TB dynamics, we do need a mathematical model to combine all of these sources into a simple framework for interpretation. There has been a considerable amount of modeling research on TB dynamics not including HIV/AIDS [17–22]. After HIV/AIDS was recognized as a crucial factor for TB, many modeling papers focused on the effect of HIV/AIDS on TB epidemiology at the population scale [10–12, 23–28]. However, none of the models cited in this paper includes all three aspects: HIV status (HIV+/HIV-), form of TB (pulmonary TB /extra pulmonary TB), and history of previous TB treatment (new/relapsed TB cases). As a result, it is difficult to apply these models for quantitatively analyzing our data sets. Thus, we modified the model presented by [20] to describe TB transmission in the presence of hyper-susceptible individuals in the population. Unlike the other HIV/TB modeling studies, the model developed in this study ignores the HIV transmission and HIV progression process. Furthermore, we used the AIDS prevalence to depict the dynamics of hyper-susceptible individuals in the population because [29, 30] indicates that about 90% of co-infected patients have CD4 cell count per μl less than 350–400 and the risk of TB increases exponentially with the decline of CD4 cell count. After that, we estimate parameters of the model by fitting this model to TB data collected from the NTP.

## Methods

### Tuberculosis transmission model (TTM)

For an uninfected individual, TB exposure results in either active TB (approximately 10% of the time) or latent TB (approximately 90% of the time); latent TB is defined as the state in which persistent immune response to Mycobacterium tuberculosis antigens but not clinical evidence of active TB exists. It is also possible for a patient with latent TB to develop active TB due to a new infection. This process is called *exogenous reinfection.* Note that TB exposure may cause the phenomenon in which mycobacteria remains in lungs and is not transmissible. The term *endogenous reactivation* describes the process in which these mycobacteria are activated, leading to active TB in an individual with a previously latent infection. Individuals who are experiencing active TB for the first time are classified as new cases. For every subsequent time they experience an active TB infection, individuals are classified as relapsed cases. Successful TB treatment eliminates all TB and its clinical symptoms, and stops transmission. However, there is no microbiological testing available to confirm that all mycobacteria are completely killed in the body of TB patients after successful TB treatment. Therefore, it is possible for individuals who have recovered from active TB to be infectious with TB again in the future due to both exogenous reinfection and endogenous reactivation.

**Fig. 1:**
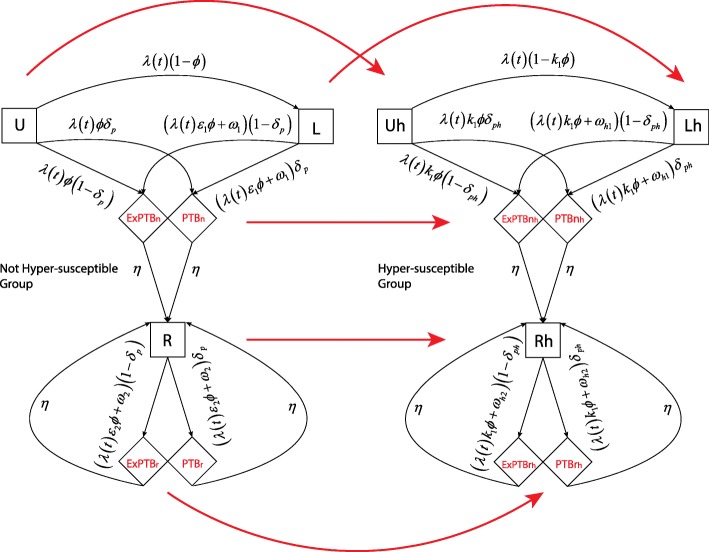
Tuberculosis transmission model. The capital letters *U*, *L*, and *R* stands for uninfected, latent, and recovered. The notations *PTB* and *ExPTB* correspond to active pulmonary TB and extra-pulmonary TB respectively. In order to distinguish new and relapsed cases, the subscripts *n* and *r* are used. The subscript *h* is used for hyper-susceptible individuals. The red arrows show the hyper-susceptibility progression process by which individuals move from group G1 to group G2

We modified the model presented by [20] to describe TB transmission in HCMC. The model used in our study is shown in Fig. 1. Hyper-susceptibility is taken into account in our model by dividing the whole population into two groups: the *not hyper-susceptible* (G1) group and the *hyper-susceptible* (G2) group. In this model, we assumed that infectivity of extra-pulmonary TB cases is negligible, so transmission occurs due to effective contact with pulmonary TB cases only. For the G1 group, people stay in uninfected class (*U*) from the time they were born. In this class they are subject to the force of infection *λ(t)*. When individuals acquire a new infection, they have probability *Φ* to develop active TB, and probability *1- Φ* to stay in the latent state (*L*). People in the latent class (*L*) can develop active TB through both exogenous reinfection or endogenous reactivation. An active TB case has probability δ_*p*_ to develop pulmonary TB (*PTBn*), and *1-* δ_*p*_ to develop extra-pulmonary TB (*ExPTBn*). After recovery, individuals move to the recovered class (*R*). In the recovered class (*R*), similar to people in the latent class (*L*), people can become infectious through exogenous reinfection or endogenous reactivation. Relapsed pulmonary and extra-pulmonary TB cases go to the *PTBr* and *ExPTBr* states respectively. For G2 group, a similar diagram is used. We assume that there are no protection due to previous TB infection among people in G2. Individuals who had active TB while they were HIV-uninfected are classified as relapsed TB if it occurs after HIV infection. Furthermore, we assume that the process that people move from G1 to G2 happens to be uniform across TB states. The difference in exogenous reinfection and endogenous reactivation between G1 and G2 groups can be seen from the meaning of hyper-susceptibility- related parameters which are listed in Table 1. Because the TB dynamics is very slow [17, 31], data collected for couple decades are not enough for distingquish between infection and reactivation processes. Therefore, in order to to improve the identifiability of our analysis, we fix ε_1_ and *ω*_*1*_ in advance. For reporting purpose, we fix the expected survival time of people (*est*) in G2 group at one year. Analyses with different values of *est* are shown in supplement.

**Table 1:** Parameter summary

Prms	Meaning	Fixed	Reference	Infer	Note
*λ (t)*	TB force of infection function			NA	See Fig. 2
*ß*_*1*_	Transmission rate of the year 1996			Yes	
*ß*_*2*_	Transmission rate of the year 2015			Yes	
*Φ*	Probability that a new infection results in an active TB.	10%	[18, 20, 21]	No	
*ε*_*1*_	Relative rate that a new infection results in an active TB between people in *L* and *U*.	0.21	[53]		
*ε*_*2*_	Relative rate that a new infection results in an active TB between people in *R* and *U*.			Yes	
*ω*_*1*_	TB reactivation rate of people in *L*.	0.00098 p-yrs	[54]		The life-time risk: 5–10%
*ω*_*2*_	TB reactivation rate people in *R*.			Yes	
*k*_*1*_	Relative rate that a new infection results in an active TB case between hyper-susceptible individuals (*Ua*, *La*, and *Ra*) and uninfected people (*U*).			Yes	
*ω*_*h1*_	TB reactivation rate of hyper-susceptible individuals with latent TB (*La*).			Yes	
*ω*_*h2*_	TB reactivation rate of hyper-susceptible individuals with recovered TB (*Ra*).			Yes	
δ_*p*_	Probability of an active TB in G1 develop into pulmonary TB.	78%	HCMC Report	No	
δ_*ph*_	Probability of an active TB in G2 develop into pulmonary TB.	57%	HCMC Report	No	
*d*_*ptb*_	Death probability of PTB case.	1%	HCMC Report	No	
*d*_*Exptb*_	Death probability of ExPTB case.	10%	HCMC Report	No	
*s*_*ptb*_	Probability of successful treatment for PTB	95%	HCMC Report	No	
*s*_*Exptb*_	Probability of successful treatment for ExPTB	80%	HCMC Report	No	
*η*	Recovery rate	2		No	Average infectious time: 6 months.
*γ*	Infectivity of co-infected pulmonary cases			Yes	
*r*_*new-tb*_ *(t)*	Reporting rate of new TB cases at year*t*			NA	
*r*_*relapsed-tb*_ *(t)*	Reporting rate of relapsed TB cases at year *t*			NA	
*r*_*1*_	Parameter of *r*_*new-tb*_*(t)* and *r*_*relapsed-tb*_*(t)*			Yes	See Fig. 2
*r*_*2*_	Parameter of *r*_*relapsed-tb*_*(t)*			Yes	
*Breaking-year*	Parameter of *r*_*relapsed-tb*_*(t)* - breaking year.			Yes	
*b*	Birth rate			No	Parameter *b* is adjusted to sustain the HCMC population size GSO’s report.
*N*	Population Size		[32]	No	
*μ*	Natural death rate	0.0054	[32]	No	
*sc*	Scaling parameter	1.9508		No	See Section 1.1 of supplement
*est*	Expected survival time of hyper-susceptible individuals.	1 yr		No	

On top of birth, death, and hyper-susceptibility progression processes, we used deterministic model that is depicted by Fig. 1 to describe the transmission process of TB:

Where the force of infection is defined by:

2 $$ \lambda (t)=\beta (t)\frac{PTBn(t)+ PTBr(t)+\gamma {PTBn}_h(t)+\gamma {PTBr}_h(t)}{\mathrm{Total}\ \mathrm{Population}\ \mathrm{Size}\ \mathrm{at}\ \mathrm{year}\ t} $$

### Demographic and hyper-susceptible individual data

In order to build and simulate the TB transmission model, the population size of G1 and G2 groups each year in HCMC are required in advance. Firstly, information about the population size of HCMC from 1993 to 2015 is derived from the General Statistics Office of Vietnam (GSO) [32]. The natural death rate of the population in HCMC is fixed at 0.0054 – the average death rate reported by GSO. The birth rate was adapted to maintain the population size as GSO reports. Second, because we assume the number of AIDS is representative for the number of individuals in G2 group, the reported number of new AIDS cases of Vietnam from 1993 to 2015 is collected from the Vietnam Ministry of Health [9, 33]. Note that due to lack of data for the year 2014, the reported number from the year 2014 is interpolated by averaging this number of the year 2013 and 2015. Then, the number of individuals in G2 group in a given year is reconstructed using the four following assumptions:

1. The number of AIDS cases is representative of the number of individuals in G2 group.
2. The number of annual newly AIDS cases in HCMC is proportional to that number in Vietnam.
3. The expected survival time of people in G2 group is constant over time.
4. The population size of G2 group in HCMC in 2015 is 19,973 as estimated by Vietnam’s HIV program [9].

Only three first assumptions are needed to identify the shape of G2’s population size curve. Then, we employ scaling parameter (*sc*) to adjust this curve to satisfy the fourth assumption. The number of individuals in G2 group in any given year follows after that. Further details of how these four assumptions are implemented is shown in Section 1 of supplement.

### Tuberculosis data

We fit the model to three sources of data. Firstly, annual TB notification data from HCMC are collected from the Annual Tuberculosis Report from 1996 to 2015. This report is the combination of quarterly reports from all 24 DTUs in HCMC and Pham Ngoc Thach hospital, a national referral hospital for lung diseases and tuberculosis. Location of collecting sites are shown in Additional file 1: Figure S7. In summary, each annual report includes the number of new and relapsed pulmonary TB and total extra-pulmonary TB cases treated at the DTUs in the corresponding years. From 2009, extra-pulmonary TB cases were also classified into new and relapsed. Because pediatric TB is under-reported in Vietnam [34], more than 95% of reported cases of this data set are from adult. We call this data D1.

Secondly, blood samples are collected from 78 Vietnamese healthy subjects working at Oxford University Clinical Research Unit in HCMC and Interferon-Gamma Release Assays (IGRAs) are used to examine the history of TB exposure. These subjects have never been diagnosed as having active TB. IGRAs measure Interferon-Gamma produced by T lymphocytes in response to TB antigens. The assays are employed to identify latent TB in healthy volunteers who are in working age. In total, 46/78 (59%) of these subjects is tested positive and we adopt this as an estimate of the proportion of latent TB in HCMC in this work. Since the sensitivity and specificity of this test are over 96% [35], we do not consider the type 1 and type 2 error of the test. We call this data D2.

Thirdly, in order to investigate the prevalence of hyper-susceptible individuals among TB patients, we collect the HIV status of 1000 random TB patients in HCMC each year from 1996 to 2014 from patient records of all districts. Further information about those patients are anonymous. We call this data D3.

### Simulation and maximum likelihood estimation

The model fitting method that is employed in this paper is maximum likelihood estimation. In order to compute the likelihood of a parameter set, we simulate the system depicted by eq. (1). After that, we compute the likelihood value of this parameter set. We use standard simplex method [36] in GSL library of C++ for the maximum likelihood estimation. The detail of simulation and maximum likelihood method is shown in Section 1.2 and 1.3 of supplement.

### Hypothesis testing

**Fig. 2:**
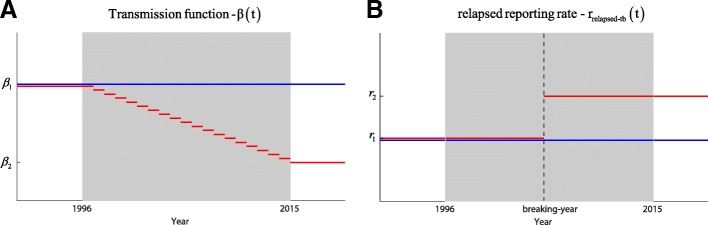
Hypothesis of contact parameter and relapsed reporting rate. The grey area shows the period TB data were collected. In panel (**a**), the blue line and red line represent two different scenarios on transmission rate function *ß(t).* While the blue line stands for the case in which transmission function is constant over time, the red line shows the case where this function is assumed to be piece-wise and linearly decreasing (or increasing) from 1996 to 2015. In total, two parameters at most (*ß*_*1*_ *and ß*_*2*_) are employed to parameterize for the transmission function. In panel (**b**), the blue and the red line represent two different hypotheses of relapsed reporting rate *r*_*relapsed-tb*_*(t).* The blue line corresponds to the case in which reporting rate of relapsed cases is constant over time. The red line corresponds to the case that the relapsed reporting rate changed over time. Note that the incidence reporting rate *r*_*new-tb*_*(t)* is always assumed to be constant over time, and equal *r*_*1*_. Therefore, in total, three unknowns at most (*r*_*1*_, *r*_*2*_, and *breaking-year*) are used to describe both *r*_*new-tb*_*(t)* and *r*_*relapsed-tb*_*(t)*. The *breaking-year* just takes discrete value from 1996 to 2015

In order to exloring the TB epidmiology scenario of HCMC, we investigate two additional hypotheses about the pattern of TB transmission and reporting of relapsed TB over the past 20 years by fitting the model described by eqs. (1) to HCMC’s TB data. These two hypotheses are shown in Fig. 2. We have four different scenarios of TB transmission and reporting rate of relapsed TB:

1. Both contact parameter and reporting rate of relapsed TB are constant over time.
2. The contact parameter is time-varying and relapsed TB reporting rate is constant.
3. The contact parameter is constant and relapsed TB reporting rate is time-varying.
4. Both contact parameter and relapsed TB reporting rate are time-varying.

Hereinafter, we use the word *hypothesis* to refer to one of these four scenarios (enumerated from *H1 to H4*). The Akaike information criterion (AIC) is used for comparing different epidemiological scenarios [37].

## Results

### Epidemiological hypothesis selection

**Fig. 3:**
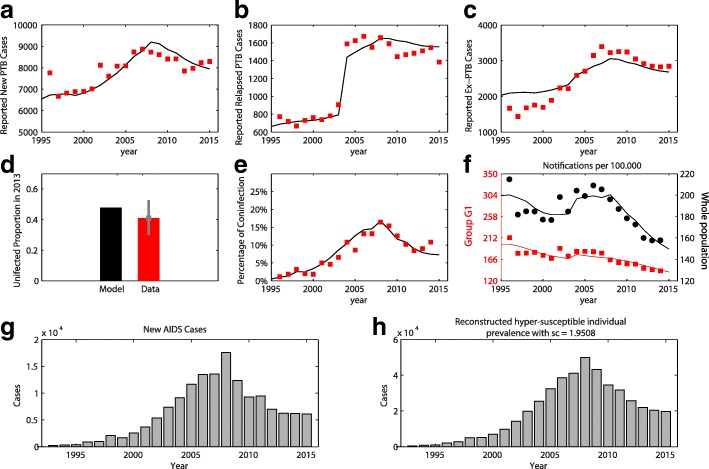
Model fit under hypothesis H4. In panels (**a**, **b**, **c**, **e**, and **f**), he dots (red and black) are the data. The continuous lines (red and black) show the reconstructed dynamics using parameters estimated by Maximum Likelihood Estimation (MLE). In panel (**d**), the black and red bar shows the uninfected proportion inferred from our model and IGRA data respectively. The gray bar shows the confidence interval of uninfected proportion computed from our IGRA data with assumption of binomial distribution. In panel (**f**), the red and black graphs (correspond to left and right y-axis) show the TB notifications of G1 group and the total population respectively. The TB notifications of G1 group are inferred by combining data set D1 and D3. Panel (**g** and **h**) shows the AIDS data and reconstructed hyper-susceptible individual curve

The summary of epidemiological hypothesis selection is shown in Table 2. The hypothesis H4 are more likely than the others by AIC. This hypothesis assumes that contact parameter declines linearly from 1996 to 2015 and the reporting rate of relapsed cases is time-varying (Table 2). According to this hypothesis, the estimate of contact parameter dropped from 37.8 in 1996 to 31.1 in 2015 (about 18% reduction) and the reporting rate of relapsed cases reached over 95% after 2003 (Table 3). Other hypotheses tend to fail in explaining either the DTU’s reporting data or IGRA data (Additional file 1: Figure S2 –S4). The AIC difference between H4 and others is at least 385 units (Table 2). Therefore, we use H4 for presenting goodness of fit in Fig. 3. Goodness of fit for other hypotheses can be seen from Additional file 1: Figure S2 to S5.

**Table 2:** Summary of AIC comparison of different epidemiological hypotheses

Hypothesis	Assumption		#optimized parameters	AIC – min(AIC)
	*ß(t)*	*r*_*relapsed-tb*_ *(t)*		
H1	*Constant*	Constant	8	1886.96
H2	Time-varying	Constant	9	385.16
H3	Constant	Time-varying	10	1787.5
H4	Time-Varying	Time-Varying	11	**0.0**

The quantity AIC – min(AIC) of the best hypothesis is zero (in bold)

**Table 3:** Estimate and 95% confidence intervals of parameters in H4. Because *breaking-year* takes discrete values, the confidence interval of this parameter is skipped

Prms	MLE	95% CI
ß_1_	37.8	[36–40.1]
ß_2_	31.1	[29.7–32.7]
ε_2_	0.37	[0.29–0.45]
ω_2_	0.0017	[0.0014–0.0022]
k_1_	0.42	[0–10]
ω_h1_	0.152	[0.09–0.163]
ω_h2_	0.06	[0.01–0.09]
γ	0.15	[0.06–0.25]
r_1_	0.57	[0.54–0.61]
r_2_	0.99	[0.96–1]
breaking-year	2003	NA

### Goodness of fit

Because there are only seven years (from 2009 to 2015) in which extra-pulmonary TB cases were classified as new or relapsed, we merge new extra-pulmonary and relapsed extra-pulmonary groups as Ex-Ptb for reporting the goodness of fit. As can be seen from Fig. 3, as the model relies on the annual counts of new hyper-susceptible individuals, the reconstructed TB dynamics is not smooth. However, it can capture qualitatively the pattern of collected TB data as both reconstructed dynamics and data peaked in 2007–2008. The panel B shows that the model captures the large increase of reported cases that was observed in 2003. In panel C, we see that the reconstructed dynamics of extra-pulmonary TB fit the data less well. The underlying reason is that we fixed the probabilities of developing extra-pulmonary and pulmonary TB in both G1 and G2 groups (δ_p_ and δ_ph_) while these numbers were reported to be year-varying. This phenomenon likely comes from variation in skill of lab technician and sensitivity of mycobacteria detection overtime in DTUs rather than any relation to TB epidemiology change. In panel D, the un-infected group (without active TB history) is estimated to be about 40% of the population which is consistent with the IGRA data. The dynamics of co-infected patients are captured neatly by the model as shown in panel E. Panel F shows the goodness of fit by plotting data and reconstructed dynamics in per 100,000 form. The number of active TB cases per 100,000 among people in G1 group decreased from 1996 to 2005. In contrast, the number of active TB cases per 100,000 for the whole population follows a different trend. After 2002, this number increased, and peaked in 2008. It suggests that despite the increase of TB in the population, the risk of active TB among people in group G1 decreases in time.

### Hyper-susceptibility and risk of active TB

As can be seen from Table 3, the confidence intervals (CIs) of *k*_*1*_, the parameter used to describe the relative rate of developing active TB due to exogenous infection process between uninfected class (*U*) and G2 group (*U*_*h*_*, L*_*h*_*,* and *R*_*h*_), are wide. The lowerbound of these CIs are zero. It indicates that the decrease in (re-)infection can be compensened by increase in reactivation. In other words, these two processes among individuals in G2 are not distinguishable from each other.

Another striking point shown in Table 3 is the risk of developing TB among people in G2. The reactivation rate of class Rh (*ω*_*h2*_) is estimated to be lower than that of class Lh (*ω*_*h1*_). Remember that we assume that the infection process is identical for individuals in Uh, Lh, and Rh. Therefore, our model suggests that people in Rh is more protected (in term of active TB) than in Lh. However, this is not the case for people in G1 group. The reactivation rate (*ω*_*2*_) of people in R is estimated to be stable, at about 0.0017 person-years, and is estimated to be consistently higher than reactivation rate (*ω*_*1*_) of people in L. Furthermore, because the relative rate of class R (*ε*_*2*_) is estimated higher than that of class L (*ε*_*1*_), the exogeneous (re-)infection process appears to have more impact on R class rather than L class. In total, it indicates that for people in group G1, the risk of relapsed active TB is higher than the risk of new active TB.

### Infectivity of hyper-susceptible TB patients

It is important to notice that the estimate of co-infected pulmonary case’s infectivity (*γ*) is low. It reveals that the contribution of co-infected patients to TB transmission in G1 is limited.

## Discussion

We have constructed a simple mathematical model to describe the TB dynamics in a population containing hyper-susceptible individuals. We consider four different epidemiological hypotheses with different assumptions about the trends in the contact parameter and reporting rate. Under these hypotheses, we fitted the model to TB data collected from HCMC. We employed AIC to identify the most likely epidemiological hypothesis occuring in HCMC from 1996 to 2015.

In summary, based on the AIC, the analysis presented in this work shows that the contact parameter of TB in HCMC reduced by around 18% from 1996 to 2015. This analysis also indicates that the reporting rate of relapsed TB patients to National Tuberculosis Control Program changed significantly to more than 95% after 2003, under the policy in which TB patients with successful treatment are asked to return for TB examination. The overall reporting rate ranges from 58 to 66%. The annual incidence is estimated at about 230 cases per 100,000. Furthermore, our estimate shows that the infectivity (*γ*) of an active TB cases with hyper-susceptibility is consistently low.

This study illustrates how HIV/AIDS drives TB dynamics in HCMC. Firstly, the wide spread of HIV/AIDS from the beginning of 1990s resulted in the increase of hyper-susceptible hosts in HCMC’s population. Consequently, the annual TB notifications in HCMC increased sharply from 1996 to 2008. This number peaked in 2008 – the year that co-infected patients accounted for about 15% of annual TB notifications. After this year, the number of TB notifications followed a decline as HIV/AIDS dynamics started to go down. Co-infected patients accounted for about 7% of TB notifications in 2015. However, after removing the impact of HIV/AIDS on TB, the Panel F Fig. 3 indicates that the annual TB notifications among people in group G1 declined from 1996 to 2015 consistently. Especially, during the growth phase of HIV/AIDS epidemics (from 1996 to 2008), the risk of active TB for people in group G1 went down despite the fact that there were more TB cases in the population (because of HIV/AIDS). Note that exogeneous (re-)infection is the main pathway in which active TB develops in G1 group. It indicates that the TB transmission (of G1) decreased in time which is consistent with hypothesis H4 – the most likely epidemiological hypothesis. Furthermore, it also implies that co-infected patients appears to have small impact on individuals in G1 group. As a result, estimate of the infectivity (*γ*) of co-infected patients is low (Table 3). In other words, the role of co-infected patients to TB transmission process in HCMC is quite limited. At this point, it is unclear that low infectivity *γ* is due to the hyper-susceptibility or the contact pattern between individuals in G1 and G2. However, it suggests that the increase in annual TB notifications per year observed from 1996 to 2008 in HCMC is inevitable when, as occurred, the number of hyper-susceptible individuals increased faster than the decrease of TB transmission rate. The sharp decrease in TB notifications observed in HCMC from 2008 to 2015 is the combined result of the decrease of TB tranmission rate and the decrease of G2’s population size.

The limited effect of HIV/AIDS to the trend of TB in people in G1 is quite consistent with [11]. However, the difference in underlying mechanism should be mentioned. The [11] suggests that limited interaction between HIV/AIDS comes from the difference between slow and fast dynamics of TB and HIV/AIDS respectively. Our model suggests that the underlying mechanism is low infectivity of co-infected patients. This indicates that how we model TB in group G2 has almost no impact on the trend of TB among people in G1. The low infectivity may be the result of contact pattern of the population. If this is a cases, there is a small sub-population (such as low income people) in which both HIV/AIDS and TB more co-circulate, and this sub-population is quite isolated from the whole population (in term of active TB). It is not necessary for these two populations to have the identical forces of infection as we model (in term of TB). It indicates that modelling for co-circulation of HIV-TB is problematic as there are many unknowns about contact pattern of this sub-population.

One important papers that is related to this work is [38]. This paper showed that latent TB is likely independent of HIV status in HIV high-risk group in Mexico. Therefore, the MTB prevalence among HIV positives is likely determined by the contact pattern of high-risk groups rather than HIV infection. Although the MTB prevalence among HIV positives is unknown in HCMC, we admit that our assumption about a well-mixed population may not reflect the TB transmission among people in G2 group. It may be the case that both HIV and TB more co-circulate a specific group (such as low income people [39–42]) in HCMC. At this point, the force of TB infection imposed to people in G2 group should be modelled as *s.λ(t)*. The parameter *s* in this situation represents for the force of TB infection enhancement. Even in the situation that the enhancement is absent (*s = 1*), the 95%CIs of *k1* is huge. The lower bound is close to zero. In other words, if we assume that the (re-)infection process has no impact on hyper-susceptible people, we still can explain our data by the (re-)activation process. This points out that introducing a new parameter (*s*) to transmission process of people in G2 does not improve the identifiability of the analysis. This argument suggests that (re-)infection process and (re-)activation process confounds each other among people in G2. Therefore, whether the trend of the (re-)infection process among people in G2 is going down like in G1 is difficult to verify from this analysis.

The enhancement of force of TB infection among people in HIV high-risk group (*s > 1*) intuitively reduces the estimate of *ω*_*h1*_ and *ω*_*h2*_. In case that *s* is fixed at 1.5, the estimate of reactivation rate of people in Rh (*ω*_*h2*_) is still significant lower than reactivation rate in Lh (*ω*_*h1*_) (Section 6 and Additional file 1: Table S3 in supplement). In other words, it is likely that people in Rh are still more protected than Lh despite the fact that our model may not give reliable evaluation of (re-)infection and (re-)activation processes among people in G2. We have to emphasize that if people in Rh are more protected than people in Lh does require further investigation and verification with a different model and data set that at least includes information about CD4 cell count and HIV progression. Several papers [30, 43, 44] indicate that the risk of TB increase after CD4 cell counts starts to decline. Therefore, modeling for a short period of HIV infection in this paper overestimates the TB risk of people in Lh. If the risk of TB were very high in early stages of HIV infection, our assumption that AIDS is representative for TB hyper-susceptible people is unrealistic. In this situation, the people in Lh would be more protected than people in Rh. However, if this were the case, we would observe many TB cases with high CD4 cell count that was contradicted by [30, 44] that showed that about 90% of co-infected patients have CD4 cell count lower than 400 cells/μl and the risk of TB increases exponentially with the decline of CD4.

If that people in Rh is more protected than Lh is the case, we hypothesize that the underlying reason is the use of ART in HCMC’s population. Much work [11, 15, 45–47] showed that widespread use of ART has strong beneficial impacts on TB mortality and morbidity in the population. The positive impact of ART on TB dynamics is due to ART preventing the decline of CD4 cell count among HIV positives. In Vietnam, ART use started in 2004. From 2004 to 2015 in Vietnam, according to the HIV guidelines, only HIV infected people in clinical stage 3 and 4 were recommended for ART (See Section 2, 3, 4, and 5 of supplement). Once an HIV positive individual starts to use ART, they continue to use ART for the rest of their life. Therefore, for individuals in Uh and Lh, it is unlikely that they have used ART before. Many people start their HIV treatment at the time they are diagnosed with active TB. For the individuals in Rh, it is likely that they had already started their HIV treatment before. Hence, they have greater protection against relapses of TB. Therefore, the delay in HIV treatment may impose a considerable TB burden on HCMC which could be avoided by starting ART earlier, and the number of TB cases in HCMC benefited little from the HIV program from 1996 to 2015.

One limitation of our work is the expected survival time of people in G2. Although this parameter is shown to have considerable variation within the population, the *est* is assumed to be homologous throughout the analysis. The heterogeneity of *est* comes from variation in the kind of high risk groups, the type of opportunistic infection, and especially ART status. However, these kinds of information were not recorded in our data. Another limitation of our work is how we simulate our dynamical system. Because the expected survival time of people in G2 group is relatively short, year by year simulation in this work may result a crude approximation of TB dynamics. One improvement is that instead of using year-to-year simulation, month-to-month simulation could be applied. However, it is important to emphasize that month-to-month simulation requires that the hyper-susceptibility data set is available by month which is a the limitation of the available data. Misclassification of TB patients is also a disadvantages of the model. It is believed that misclassification always happens and varies in time as the community awareness and TB diagnosis technique are improved. Nevertheless, the model is not designed to capture this fact. Thus, it may bias the reporting rate and reactivation rates.

One criticism that can be made about our TB transmission model is the assumption that the average infectious period of all TB patients is only six months. The infectious period of a TB patient is defined as the period that this patient can spread TB to others. Note that the behaviour of our TB model may not change if we decrease the infectious period and increase the transmission parameter at the same time. Therefore, our analysis appears to be robust with respect to this parameter. In the literature, there is a significant variation of TB infectious period used. The paper [20] suggest that this period is six months that equals to the duration of TB treatment. Another example can be seen in [48]. This paper suggests that period of infectivity (of adult) ranges from 30 days to 120 days. The paper [21] suggest this period is two years. For untreated TB patients, the paper [49] assume this duration is five years. However, this quantity in [10] is fixed at two years. The paper [50] does systematic review and conclude that this period is about 3 years (with very high mortality rate). From clinical viewpoint, this period for TB should be considered as the period that TB patients have symptoms such as coughing. It is likely that patients without symptoms is believed to have very low infectivity. This period is fixed at six months in our analysis through two data set. First, the clinical data (unpublished) of TB treatment in Vietnam indicates that more than 95% TB patients are smear-negative and symptom-free after three to four months of treatment. Second, the delay time for finding TB treatment since symptom appearance in HCMC about 43 days (see Additional file 1: Figure S6). Further investigation on infectious period may shed new light to the relationship between this factor and the persistence of TB in the population.

Another criticism is about the population-representativeness of our data sets. Overall, pediatric TB is under-reported in Vietnam [34]. Majority of reported cases used in this study (data set D1) is also from adult. Consequently, whether the prevalence of HIV among TB patients (data set D3) is population representative is unclear. Furthermore, the data set D1 just records TB patients in public sector only (We used this number as *TB notifications*). TB patients that receive similar TB treatment in private sector are not recorded. The number of TB patients in private sector is believed to be considerable. Moreover, the IGRA data set D2 – a small data set with only 78 samples - was collected from people in working age. One important point is that we have to emphasize that after comparing the estimate of true incidence and our reporting data set D1, the proportion of active TB cases that goes to public sector in HCMC for TB treatment ranges from 58 to 66% which is very consistent with the estimate presented by WHO [7, 51] for Vietnam (about 65%). In other words, this model likely reflects the true TB dynamics in HCMC. At this point, it is unclear that if the model only represents the adult population or the whole population.

Although this paper shows that the TB transmission of HCMC was going down from 1996 to 2015, we need to mention some confounding factors. In order to describe the change of the transmission process, we assume that the contact parameter is time-varying. However, the change in infectious time of TB patient (we do not have data on this factor) may reduce the TB prevalence and give the same result. In general, the TB dynamics is very slow [17, 31] compared to other acute infectious disease such as influenza or hand foot mouth disease. One TB epidemics may last for a couple centuries [17]. Thus, any changes in non-linear terms (such as contact parameter) can be approximated by changes in linear term (such as reactivation rate or infectious period) for a couple of decades. For this reason, it is unclear the underlying mechanism of the declining TB transmission. Over the past 20 years, there was a significant change in all aspects of social economy that may have positive impact on TB transmission in HCMC. For example, the Vietnam government decided to open the country and changed from communist economy to market economy. The Gross Domestic Product (per capita) of Vietnam changed from 323 USD in 1996 to 2171 USD in 2015 [52]. People spend more money on health care. Furthermore, the awareness of people about TB also changed as the NTP ran many public engagement campaigns and adopted new policies to educate people. The urbanization may also have positive impact as it reduced the over-crowding in big cities such as HCMC. Furthermore, in order to keep our analysis simple, we assume that the TB dynamics in 1992 was endemic and the contact parameter varied linearly from 1996 to 2015. However, these assumptions may be violated in reality. If this is a case, this violation may have negative impact on our estimate of TB transmission reduction quantitatively.

The main focus of this paper is evaluating the impact of HIV/AIDS to TB dynamics in HCMC. In order to achieve this target with our data set, we constructed a mathematical model with very simple HIV/AIDS structure. Note that our result shows that HIV/AIDS has limited effect on TB transmission in population which is consistent with [11]. Although this result indicates that the simple HIV/AIDS structure of our model appears not to have negative impact on the main target of the paper, it is important to emphasize that this simple structure does not give us a satisfied answer on the TB epidemiology of hyper-susceptible individuals. The risk of active TB increases exponentially with CD4 cell count reduction [29, 30]**.** Therefore, assuming this risk is homologous among hyper-susceptible individuals and using only people with AIDS to depict the hyper-susceptible individuals may not give us a full picture of TB infection and reactivation among HIV positives. As a result, some findings of this paper can serve as new hypotheses for further investigating TB epidemiology of hyper-susceptible individuals in a suitable model with better HIV/AIDS structure and data.

The multi-drug resistant (MDR) topic in TB is receiving more and more interest from the scienctific community. The true mechanism of MDR in Vietnam is still a large unanswered question because the prevalence of MDR among people with and without AIDS are very different (4% and > 20% respectively). Future developments of this modeling work will concentrate on answering understanding mechanisms to explain the strong correlation between AIDS and MDR in Vietnam.

## Conclusion

This paper is the first paper that quantitatively investigates the TB dynamics of HCMC using a mathematical model to intergrating many data sources. It contributes to our knowledge of TB dynamics in HCMC from 1996 to 2005 – the period in which HIV/AIDS widely spread in Vietnamese population. As the HIV/AIDS widely spread in the population, the number of hyper-susceptible individuals inevitably increased. Therefore, despite the declining trend of TB (re-)infection process among people in G1, the number of TB notifications in HCMC increased sharply from 1996 to 2008.

For co-infected patients, this paper indicates that their infectivity is very low. In other words, how we model TB dynamics in G2 has almost no impact to G1. Furthermore, this paper shows that (re-)infection and (re-)activation processes of G2 have very low identifiability. It implises that if (re-)infection process of G2 was declining from 1996 to 2015 (like G1) is impossible to verify with our model and data.The analysis in this paper suggests that to hyper-susceptible individuals, individuals in Rh are likely more protected (in term of TB) than individuals in Lh. We hypothesize that the underlying reason for this is the guidelines about timing of the start of ART. This situation requires further investigation and verification with different data sets and models. If this situation is the case, we suggest HIV treatment policy in Vietnam should be reconsidered as Vietnam is a high TB burden country.

## Additional files

Additional file 1: This includes the supplementary document for this paper. (DOCX 2715 kb)

Additional file 2: This is data set D1 of this paper. (CSV 548 bytes)

Additional file 3: This is data set D2 of this paper. (CSV 30 bytes)

Additional file 4: This is data set D3 of this paper. (CSV 372 bytes)

Additional file 5: This is the AIDS data set used in this paper. (CSV 274 bytes)

## Abbreviations

AIC: Akaike Information Criterion
ART: Antiretroviral Therapy
CI: Confidence Interval
DOTS: The Direct Observed Treatment, Short Course
DTU: District Tuberculosis Unit
ExPTB: Extra-pulmonary Tuberculosis
GSO: General Statistics Office of Vietnam
HCMC: Ho Chi Minh City
IGRA: Interferon-Gamma Release Assay
MDR: Multi-drug Resistant
MTB: Mycobacterium Tuberculosis
NTP: National Tuberculosis Control Program
PTB: Pulmonary Tuberculosis
TB: Tuberculosis
