# Supplementary material for: Modeling tuberculosis dynamics with the presence of hyper-susceptible individuals for Ho Chi Minh City from 1996 to 2015
Source: BMC Infect Dis. 2018 Oct 1;18:494. doi: 10.1186/s12879-018-3383-3 (PMC6167874; doi:10.1186/s12879-018-3383-3)
Supplement: Supplementary file 1 — This includes the supplementary document for this paper. (DOCX 2715 kb) [file 12879_2018_3383_MOESM1_ESM.docx]

**Supplement for “Modeling tuberculosis dynamics with the presence of hyper-susceptible individuals for Ho Chi Minh City from 1996 to 2015.**

**1 Methodology**

**1.1 Hyper-susceptible Individual Prevalence**

**1.1.1 AIDS Data**

The AIDS incidence data were collected from [1,2]. The missing reported AIDS incidence of the year 2014 was inferred by averaging of reported AIDS incidence of the year 2013 and 2015.

**1.1.2 Assumption**

In order to reconstruct hyper-susceptible individual prevalence of any given year, we imposed the three following assumptions:

1. The number of AIDS is representative for the number of hyper-susceptible individuals in the population.
2. The number of yearly new hyper-susceptible individuals in Ho Chi Minh City (HCMC) is proportional to that number of Vietnam.
3. The expected survival time of these individuals is constant over time.
4. The total number of hyper-susceptible individuals in HCMC in 2015 was 19,973 as represented by [2].

**1.1.3 Survival Probability**

We denote *p* as the yearly survival probability of a hyper-susceptible individual. From the definition of the expecting survival time of a hyper-susceptible individual (*est*), the relation between *p* and *est* is characterized by following formula:

|  | $est=\sum_{i=1}^{\infty} p^{i}i$ | (S1) |
| --- | --- | --- |

We denote:

|  | $f\left(p\right)=\sum_{i=1}^{\infty} p^{i}i$ | (S2) |
| --- | --- | --- |

Remember that:

|  | $\sum_{i=1}^{\infty} p^{i}i=\sum_{i=0}^{\infty} p^{i}i-p^{0}=\frac{1}{1-p}-1=\frac{p}{1-p}$ | (S3) |
| --- | --- | --- |

We apply (S3) into (S2):

|  | $f\left(p\right)+\frac{p}{1-p}=\sum_{i=1}^{\infty} p^{i}i+\sum_{i=1}^{\infty} p^{i}=\sum_{i=1}^{\infty} p^{i}\left(i+1\right)$ | (S4) |
| --- | --- | --- |

Multiply both sides by *p*, we have:

|  | $p\left(f\left(p\right)+\frac{p}{1-p}\right)=p\sum_{i=1}^{\infty} p^{i}\left(i+1\right)=\sum_{i=1}^{\infty} p^{i+1}\left(i+1\right)=f\left(p\right)-p$ | (S5) |
| --- | --- | --- |

Finally, we derive:

|  | $f\left(p\right)=\frac{p}{\left(1-p\right)^{2}}$ | (S6) |
| --- | --- | --- |

In other words, the relation between survival probability *p* and *est* is given:

|  | $\frac{p}{\left(1-p\right)^{2}}=est$ | (S7) |
| --- | --- | --- |

Because *p* ≠ 1, we have:

|  | $\left(1+p^{2}-2p\right)est-p=0$ | (S8) |
| --- | --- | --- |

Note that, this quadratic equation always has exactly one solution in between 0 and 1. Therefore, *p* can be computed easily by solving this equation and choose the solution in (0, 1) .

**1.1.4 Scaling Parameter – *sc***

As the definition of survival probability, *p* demonstrate the probability that a hyper-susceptible individual still survive for one more year. Therefore, it plays an important role in identifying how many observed hyper-susceptible individuals that will survive until a given future year. The curve that shows the relationship between number of survival observed hyper-susceptible individuals and time is proportional to the true hyper-susceptible individual prevalence dynamics by the assumption 2. Therefore, in order to identify the true hyper-susceptible individual prevalence dynamics, we scale this curve with *sc*. The value of *sc* is adjusted so that the number of hyper-susceptible individuals in 2015 is about 19,973 as assumption 4. The relation between the number of new hyper-susceptible individuals and reported new hyper-susceptible individuals (est) is given:

|  | $\#\left\{\text{new} \text{hyper-susceptible} \text{individuals}\right\}=\#\left\{\text{reported} \text{new} \text{hyper-susceptible} \text{individuals} \right\}\times sc$ | (S9) |
| --- | --- | --- |

The hyper-susceptible individual prevalence with different value of expected survival time (*est*) is shown in Figure S1. Note that in order to sustain the hyper-susceptible population in 2015 is 19,973, when the value of *est* increases the value *sc* decreases. Therefore, if *est* increases the number of new hyper-susceptible individuals per year is reduced by equation (S9).

**1.2 Simulation**

We use the following notation θ to represent our parameter set: the collection of all eleven parameters from Table 1 of the main text. In order to simulate the system with a given θ, two following stages were applied to the model:

- Stage 1: the equation system (1) of the main text is simulated (for 400 years) to make the TB dynamics is endemic. After that, this condition is used as initial condition for the year 1992 - the last year that number of hyper-susceptible individual was zero.
- Stage 2: The equation system (1) is simulated year by year from 1992 to 2015. The final condition of simulation for a given year is updated with natural birth rate, and natural death rate. Based on the value of scaling parameter (*sc*) and the expected survival time for people with hyper-susceptibility (*est*), we compute the number of new hyper susceptible individuals, number of hyper-susceptible individual deaths (see 1.1.3 and 1.1.4). After that, the distribution of these new hyper-susceptible individuals is computed. Next, we update the final condition with two processes: people progress to hyper-susceptible group from not hyper-susceptible group, and death process of hyper susceptible individuals. Then this final condition is used as initial condition for the next year. The distribution of new hyper-susceptible individuals are assumed to be uniform as following:

|  | $\begin{matrix} \#\left\{\text{U}\to\text{Uh}\right\}=\frac{\#\{\text{new} \text{hyper-susceptible}\}\times\text{U}}{\text{G1's} \text{population} \text{size}} \\ \#\left\{\text{L}\to\text{Lh}\right\}=\frac{\#\{\text{new} \text{hyper-susceptible}\}\times\text{L}}{\text{G1's} \text{population} \text{size}} \\ \#\left\{\text{ExPTBn}\to\text{ExPTBnh}\right\}=\frac{\#\{\text{new} \text{hyper-susceptible}\}\times\text{ExPTBn}}{\text{G1's} \text{population} \text{size}} \\ \#\left\{\text{PTBn}\to\text{PTBnh}\right\}=\frac{\#\{\text{new} \text{hyper-susceptible}\}\times\text{PTBn}}{\text{G1's} \text{population} \text{size}} \\ \#\left\{\text{R}\to\text{Rh}\right\}=\frac{\#\{\text{new} \text{hyper-susceptible}\}\times\text{R}}{\text{G1's} \text{population} \text{size}} \\ \#\left\{\text{ExPTBr}\to\text{ExPTBrh}\right\}=\frac{\#\{\text{new} \text{hyper-susceptible}\}\times\text{ExPTBr}}{\text{G1's} \text{population} \text{size}} \\ \#\left\{\text{PTBr}\to\text{PTBrh}\right\}=\frac{\#\{\text{new} \text{hyper-susceptible}\}\times\text{PTBr}}{\text{G1's} \text{population} \text{size}} \end{matrix}$ | (S10) |
| --- | --- | --- |

**1.3 Maximum Likelihood Estimation**

The observation process of data set D1 of the main text is assumed to have Poisson distribution as follow:

|  | $\begin{matrix} \#\left\{\text{reported} \text{new} \text{PTB} \text{cases} \text{at} t\right\} \sim Poiss\left(\#\left\{\text{new} \text{PTB} \text{cases}\right\}\times r_{new-tb}\left(t\right)\right) \\ \#\left\{\text{reported} \text{relapsed} \text{PTB} \text{cases} \text{at} t\right\} \sim Poiss\left(\#\left\{\text{relapsed} \text{PTB} \text{cases}\right\}\times r_{relapsed-tb}\left(t\right)\right) \\ \#\left\{\text{reported} \text{new} \text{ExPTB} \text{cases} \text{at} t\right\} \sim Poiss\left(\#\left\{\text{new} \text{ExPTB} \text{cases}\right\}\times r_{new-tb}\left(t\right)\right) \\ \#\left\{\text{reported} \text{relapsed} \text{ExPTB} \text{cases} \text{at} t\right\} \sim Poiss\left(\#\left\{\text{relapsed} \text{PTB} \text{cases}\right\}\times r_{relapsed-tb}\left(t\right)\right) \end{matrix}$ | (S11) |
| --- | --- | --- |

Where *r_new−tb_(t)* and *r_relapsed−tb_(t)* represent for the chance that a new active TB case and a relapsed TB case will be reported to the DTUs at the year *t* respectively. The Poisson means on the righthand sides of equations (S11) are obtained from simulating the differential equations (1) of the main text (see 1.2). The lefthand side quantity is data points of D1. For IGRA data, data set D2 of the main text, the probability of observing a positive among healthy people without history of active TB at the year 2013 is computed by simulation as follow:

|  | $p_{IGRA}\left(2013\right)=\frac{L\left(2013\right)}{L\left(2013\right)+U\left(2013\right)}$ | (12) |
| --- | --- | --- |

The number of positives among 78 IGRAs at the year t is assumed to have binomial distribution:

|  | $\#\left\{IGRA positives\right\}\sim Bino\left(p_{IGRA}\left(2013\right),78\right)$ | (13) |
| --- | --- | --- |

For the co-infection data set, data set D3 of the main text, the probability of observing an active TB case that is hyper-susceptible is also computed by simulation:

|  | $p_{\text{active} \text{TB} \text{with} \text{hyper-susceptibility}}\left(t\right)=\frac{\#\{\text{expected} \text{reported} \text{co-infected} \text{patients} \text{at} \text{year} t\}}{\#\{\text{expected} \text{total} \text{reported} \text{TB} \text{at} \text{year} t\}}$ | (14) |
| --- | --- | --- |

The number of hyper-susceptible individuals among 1000 TB patients in year t has binomial distribution:

|  | $\#\left\{\text{co-infected} \text{patients} \text{in} \text{D3}\right\}\sim Bino\left(p_{\text{active} \text{TB} \text{with} \text{hyper-susceptibility}}\left(t\right),1000\right)$ | (15) |
| --- | --- | --- |

By the assumption that three data sets (D1, D2, and D3) are independent, the likelihood function is defined as follows:

|  | $L\left(\theta\right)=p\left(D1;D2;D3\vert\theta\right)=p\left(D1\vert\theta\right)\times p\left(D2\vert\theta\right)\times p\left(D3\vert\theta\right)$ | (16) |
| --- | --- | --- |

If we take log of (S16), we have:

|  | $l\left(\theta\right)=\log\left(L\left(\theta\right)\right)=\log\left(p\left(D1\vert\theta\right)\right)+\log\left(p\left(D2\vert\theta\right)\right)+\log\left(p\left(D3\vert\theta\right)\right)$ | (17) |
| --- | --- | --- |

The Log-likelihood function l(θ) was computed through simulation and maximized over parameters in Table 1 of the main text using standard simplex method (Nelder und Mead, 1965) in GSL library of C++. In order to identify the global maximum, we repeated the search routine with 200 different initial conditions. Log-likelihood profile was used to compute confidence intervals for parameters of interest. All figures were made in Matlab R2013a (Mathworks, Natick, MA).

**2 Clinical Staging of HIV Disease in Vietnam**
• Clinical Stage 1

– Asymptomatic

– Persistent generalized lymphadenopathy

• Clinical Stage 2

– Moderate unexplained weight loss (< 10% of presumed or measured body weight)

– Recurrent respiratory tract infections (sinusitis, tonsillitis, otitis media, pharyngitis)

– Herpes zoster

– Angular cheilitis

– Recurrent oral ulceration

– Papular pruritic eruption

– Fungal nail infections

– Seborrhoeic dermatitis

• Clinical Stage 3

– Unexplained severe weight loss (> 10% of presumed or measured body weight)

– Unexplained chronic diarrhoea for longer than 1 month

– Unexplained persistent fever (intermittent or constant for longer than 1 month)

– Persistent oral candidiasis

– Oral hairy leukoplakia

– Pulmonary tuberculosis

– Severe bacterial infections (such as pneumonia, empyema, pyomyositis, bone or join infection, meningitis, bacteraemia)

– Acute necrotizing ulcerative stomatitis, gingivitis or periodontitis

– Unexplained anaemia (< 8 g/dl), neutropaenia (< 0.5 × 109/l) and/or chronic thrombocytopaenia (< 50 × 109 /l)

• Clinical Stage 4

– HIV wasting syndrome

– Pneumocystis (jirovecii) pneumonia

– Recurrent severe bacterial pneumonia

– Chronic herpes simplex infection (orolabial, genital or anorectal of more than 1 months duration or visceral at any site)

– Oesophageal candidiasis (or candidiasis of trachea, bronchi or lungs)

– Extrapulmonary tuberculosis

– Kaposi sarcoma

– Cytomegalovirus infection (retinitis or infection of other organs)

– Central nervous system toxoplasmosis

– HIV encephalopathy

– Extrapulmonary cryptococcosis, including meningitis

– Disseminated nontuberculous mycobacterial infection

– Progressive multifocal leukoencephalopathy

– Chronic cryptosporidiosis

– Chronic isosporiasis

– Disseminated mycosis (extrapulmonary histoplasmosis, coccidioidomycosis)

– Lymphoma (cerebral or B-cell non-Hodgkin)

– Symptomatic HIV-associated nephropathy or cardiomyopathy

– Recurrent septicaemia (including nontyphoidal Salmonella)

– Invasive cervical carcinoma

– Atypical disseminated leishmaniasis

Because of the fast progression from clinical stage 3 to clinical stage 4, HIV positive individuals that are in clinical stage 3 and clinical stage 4 or CD4 cell count < 350 cells/µL are classified as AIDS.

**3 HIV Treatment Guideline of Vietnam Ministry of Health – 2005**

ART should be initiated in adults and adolescents with severe or advanced HIV clinical disease in following situations:

• If CD4 cell count is available:

– Individuals in clinical stage 4, regardless CD4 cell count.

– Individuals in clinical stage 3 with CD4 cell count ≤ 350 cells/µL.

– Individuals in clinical stage 1 or 2 with CD4 cell count ≤ 200 cells/µL.

• If CD4 cell count is not available:

– Individuals in clinical stage 4, regardless lymphoma cell count.

– Individuals in clinical stage 2 or 3 with lymphoma cell count ≤ 1200 cells/µL.

**4 HIV Treatment Guideline of Vietnam Ministry of Health – 2009**

ART should be initiated in adults and adolescents with severe or advanced HIV clinical disease in following situations:

• If CD4 cell count is available:

– Individuals in clinical stage 4, regardless CD4 cell count.

– Individuals in clinical stage 3 with CD4 cell count ≤ 350 cells/µL.

– Individuals in clinical stage 1 or 2 with CD4 cell count ≤ 250 cells/µL.

• If CD4 cell count is not available:

– Individuals in clinical stage 3 or 4.

**5 HIV Treatment Guideline of Vietnam Ministry of Health – 2015**

ART should be initiated in adults and adolescents with severe or advanced HIV clinical disease in following situations:

• CD4 cell count ≤ 500 cells/µL.

• Regardless CD4 cell count:

– Active TB disease.

– HBV coinfection with severe chronic liver disease.

– Pregnant and breastfeeding women with HIV.

– Individuals in a serodiscordant partnership (to reduce HIV transmission risk).

– Individuals who injects drug.

– Women who is female sex worker.

– Men who has sex with men.

– Individuals who are older than 50 years old.

– Individuals who are living in remote areas.

**6 Extra Analysis of Force of Infection Enhancement.**

It may be the case that both HIV and TB more co-circulate a specific group (such as poor people [3–6]) in HCMC. At this point, the HIV infection will occurs among people with latent TB (L) rather than un-infected people (U). Furthermore, the force of TB infection imposed to people in G2 group should be modelled as *s.λ(t).* The parameter *s* in this situation represents for the force of TB infection enhancement.

In order to evaluating the risk of new and relapsed TB in the presence of force of TB enhancement among people in G2, we assume that *s = 1.5*. Furthermore, we assume that the process that people move from G1 to G2 is not uniform:

|  | $\begin{matrix} \#\left\{\text{U}\to\text{Uh}\right\}=\#\{\text{hyper-susceptible}\}\times u \\ \#\left\{\text{L}\to\text{Lh}\right\}=\frac{\#\{\text{hyper-susceptible}\}\times(1-u)\times\text{L}}{\text{G1's} \text{population} \text{size - U}} \\ \#\left\{\text{ExPTBn}\to\text{ExPTBnh}\right\}=\frac{\#\{\text{hyper-susceptible}\}\times(1-u)\times\text{ExPTBn}}{\text{G1's} \text{population} \text{size - U}} \\ \#\left\{\text{PTBn}\to\text{PTBnh}\right\}=\frac{\#\{\text{hyper-susceptible}\}\times(1-u)\times\text{PTBn}}{\text{G1's} \text{population} \text{size - U}} \\ \#\left\{\text{R}\to\text{Rh}\right\}=\frac{\#\{\text{hyper-susceptible}\}\times(1-u)\times\text{R}}{\text{G1's} \text{population} \text{size - U}} \\ \#\left\{\text{ExPTBr}\to\text{ExPTBrh}\right\}=\frac{\#\{\text{hyper-susceptible}\}\times(1-u)\times\text{ExPTBr}}{\text{G1's} \text{population} \text{size - U}} \\ \#\left\{\text{PTBr}\to\text{PTBrh}\right\}=\frac{\#\{\text{hyper-susceptible}\}\times(1-u)\times\text{PTBr}}{\text{G1's} \text{population} \text{size - U}} \end{matrix}$ | (S18) |
| --- | --- | --- |

Where *u* is fixed at 10% and 20%. In other words, we assume that there are only 10% and 20% of new hyper-susceptible people every year are uninfected with TB.

The result of this analysis is shown in Table S3. When *u* varies from 20% to 10% the both reativation rates (*ω_h1_* and *ω_h2_*) reduces. The value of *ω_h2_* is close to zero*.* Furthermore, it is consistent that the estimate of *ω_h2_* is lower than estimate of *ω_h1_*. The 95% CIs of *ω_h1_* exclude the 95% ICs of *ω_h2_* (except for the situation that the *est* parameter is fixed at 1 year. Therefore, in the situations characterized by this extra analysis, people in Rh are likely more protected than Lh.

**7 Figures**

**
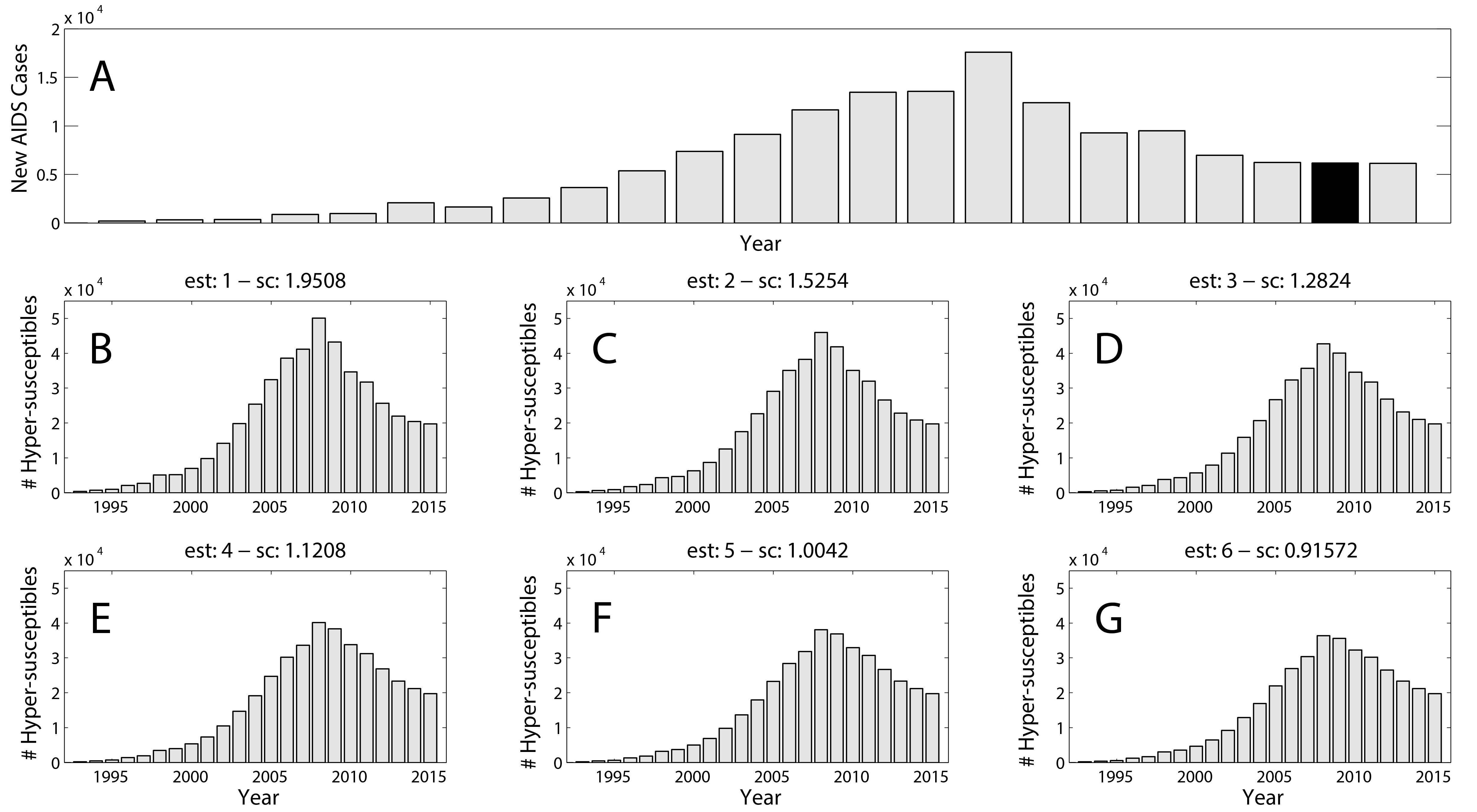
**

Figure S1: The AIDS incidence data and inferred hyper-susceptible individual prevalence. In panel A, the gray bars are data collected from [1,2]. The black bar is AIDS incidence that is interpolated by taking average of AIDS incidence of two neighbor years. In panel B, C, D, E, F, and G, the hyper-susceptible individual prevalence that corresponds to different expecting survival time (*est*) was plotted. All of graphs in B, C, D, E, F, and G are shown in the same scale.

**
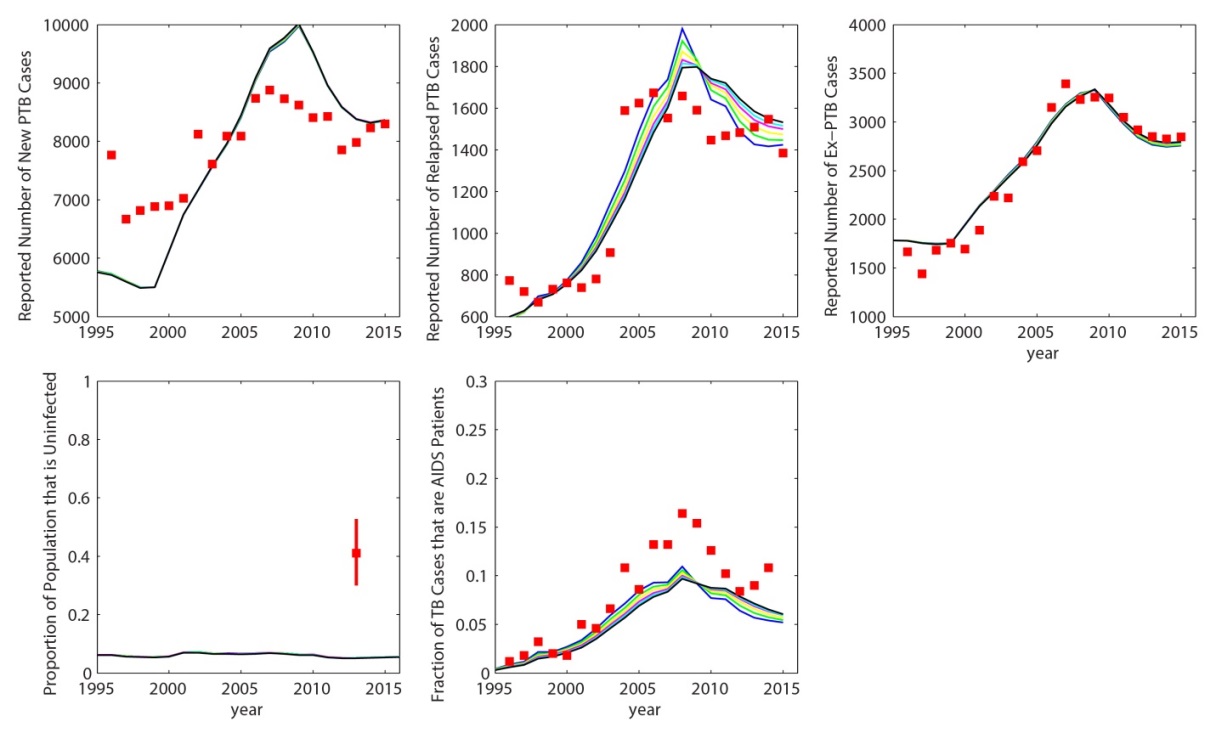
**

Figure S2: Goodness of fit under the hypothesis H1 (constant forcing function and constant relapsed reporting rate). In these panels, the red dots are the data. The red bar in bottom left panel shows the 95%CI of the proportion of un-infected class computed from IGRA data only with assumption of binomial distribution. The lines shows the reconstructed dynamics using parameters estimated from the model by Maximum Likelihood Estimation (MLE). The blue, green, yellow, magenta, cyan, and black lines correspond to the situation in which the expected survival time of individuals with hyper-susceptibility (*est*) varies from one year to six years respectively.

**
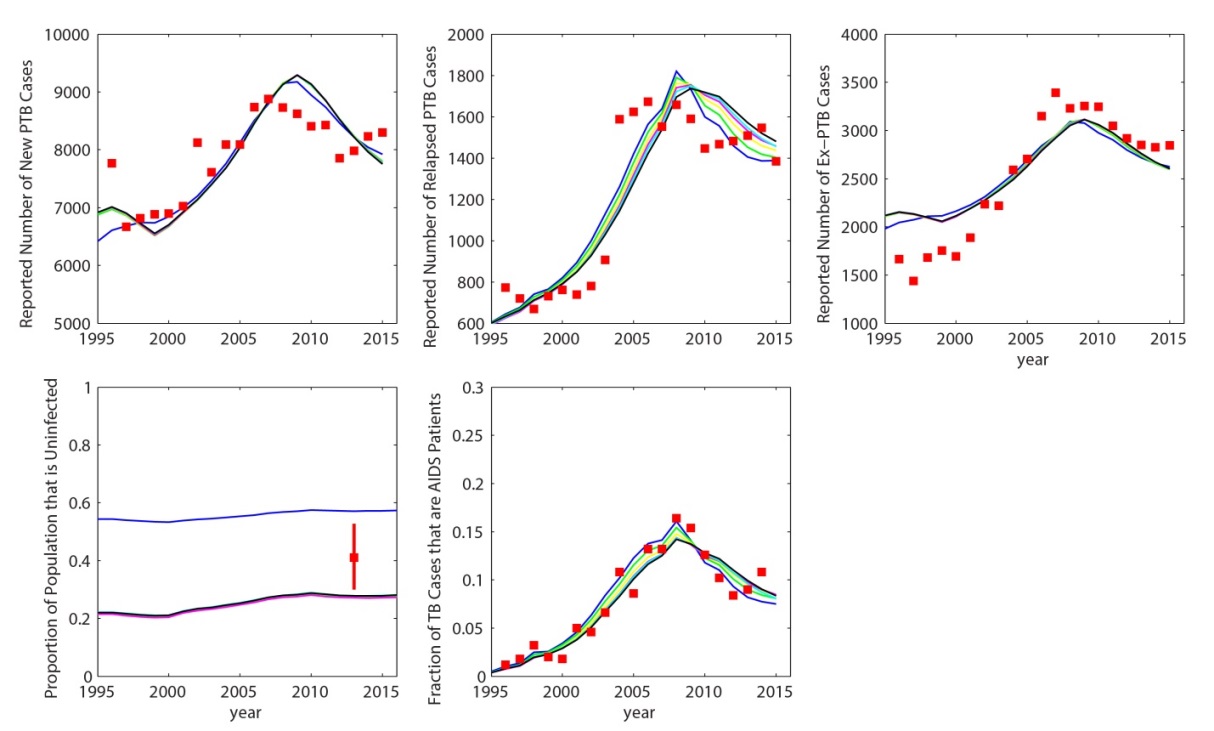
**

Figure S3: Goodness of fit under the hypothesis H2 (time-varying forcing function and constant relapsed reporting rate). Other settings are similar to Figure S2

**
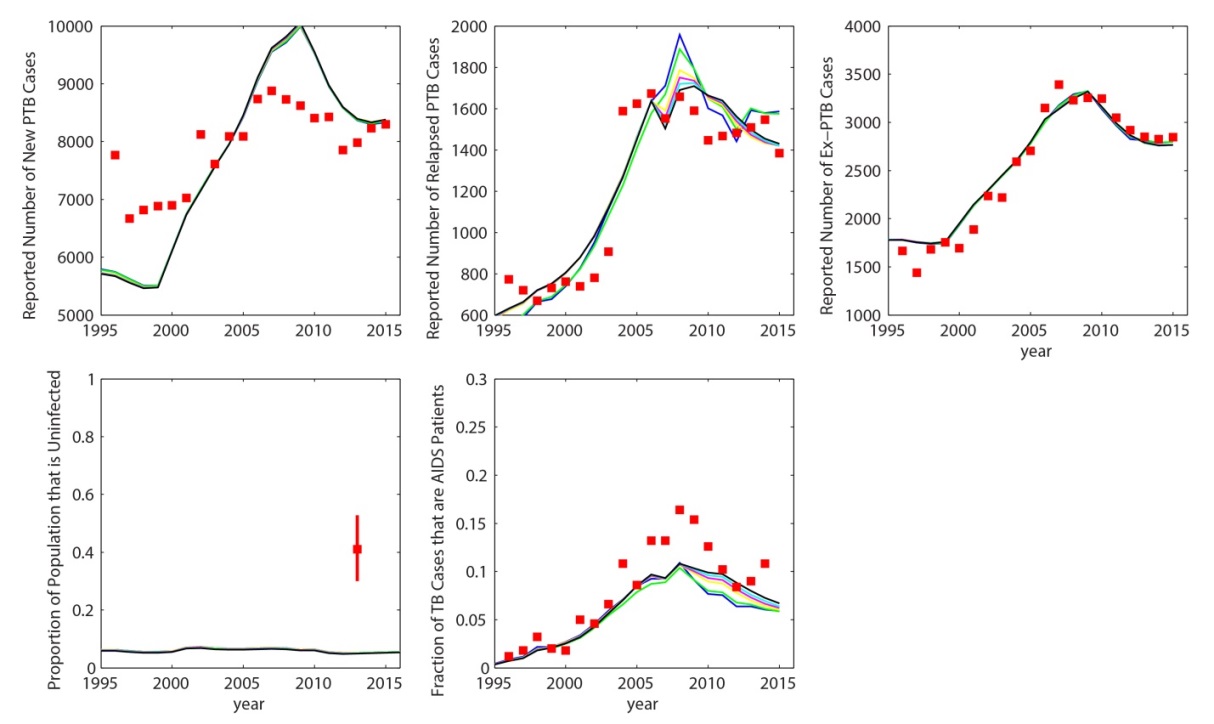
**

Figure S4: Goodness of fit under the hypothesis H3 (constant forcing function and time-varying relapsed reporting rate). Other settings are similar to Figure S2.

**
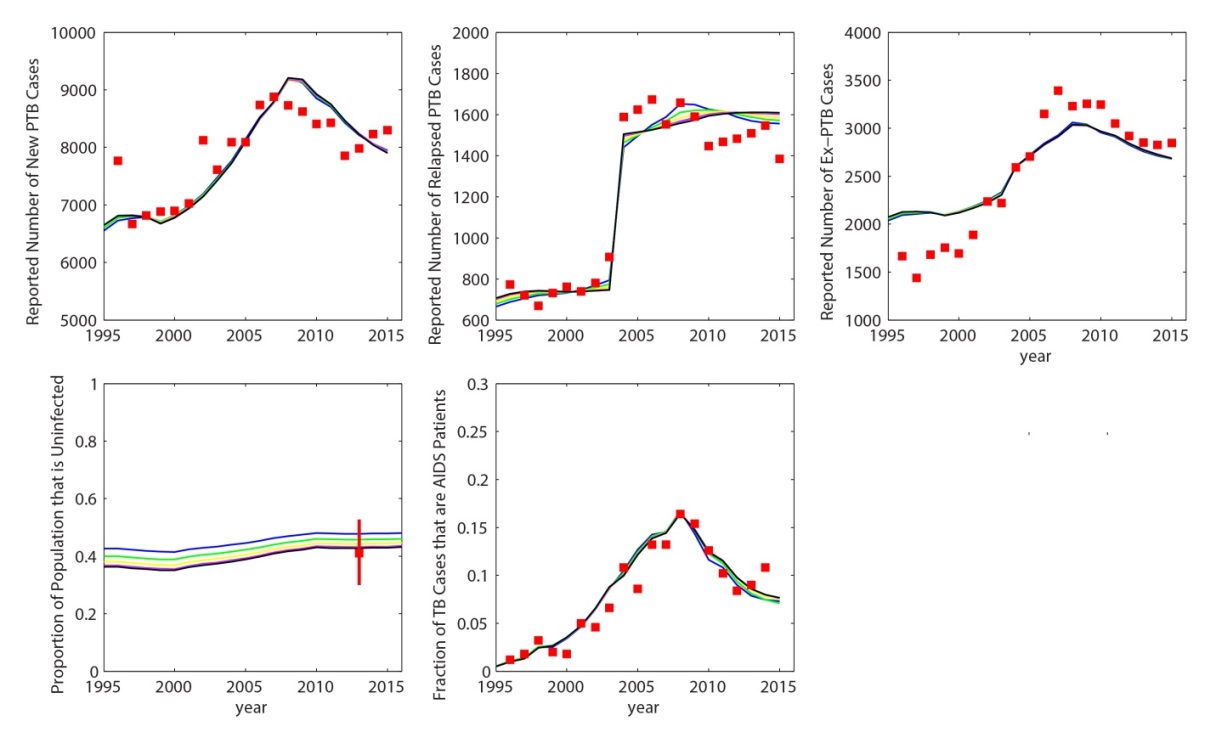
**

Figure S5: Goodness of fit under the hypothesis H4 (time-varying forcing function and time-varying relapsed reporting rate). Other settings are similar to Figure S2

**
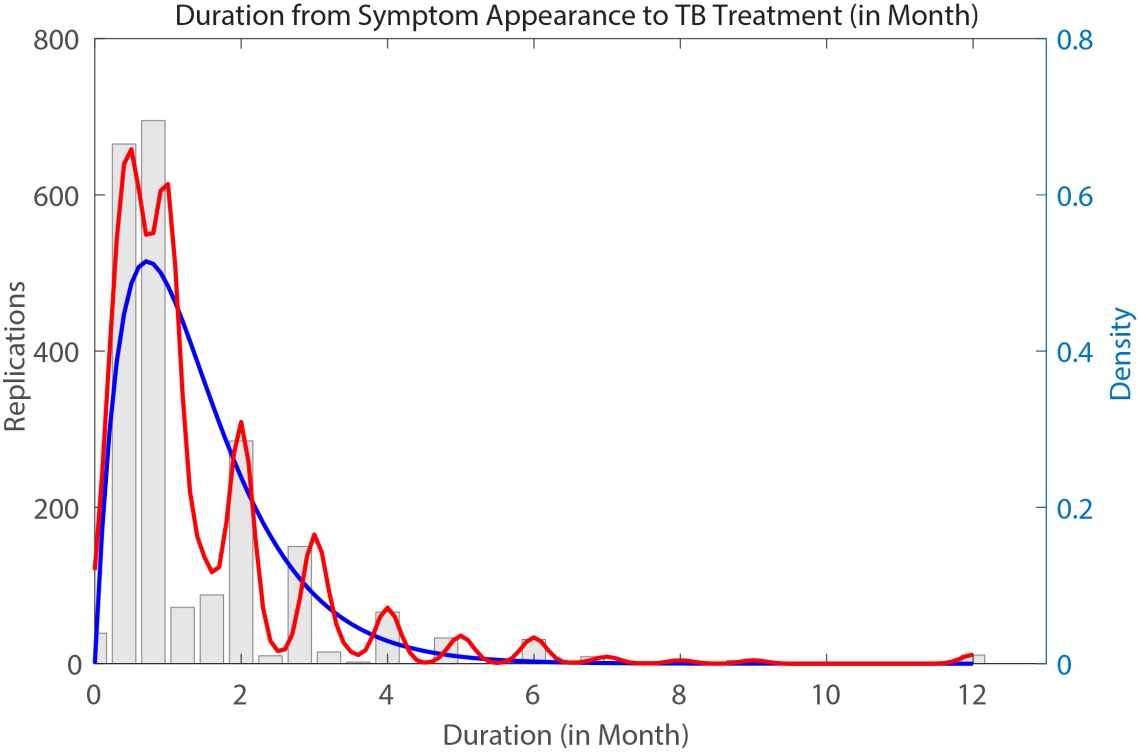
**

Figure S6: Duration from TB Symptom’s Appearance to TB Treatment (among patients with pulmonary TB). The gray bars that correspond to left y-axis are our data (collected in HCMC in 2010). The red and blue lines correspond to right y-axis. The red line is kernel smoothing density curve computed from our data. The blue line is the maximum likelihood estimation with the assumption that this duration has gamma distribution with k = 2. The mean of duration is 1.43 months (~42.8 days).


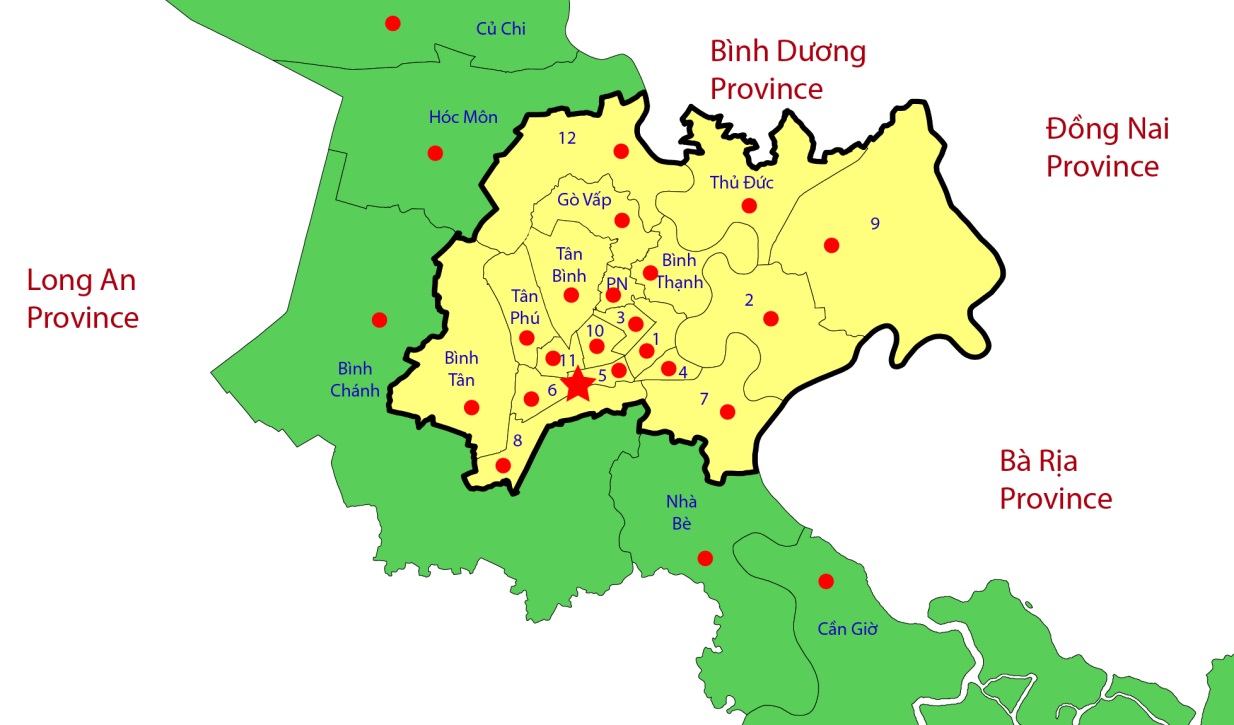


Figure S7: Ho Chi Minh City and sample collection sites. The green and yellow areas show the suburban and urban districts of HCMC respectively. The red dot in each district demonstrates the location of the DTU. The red star shows location of Pham Ngoc Thach hospital.

**
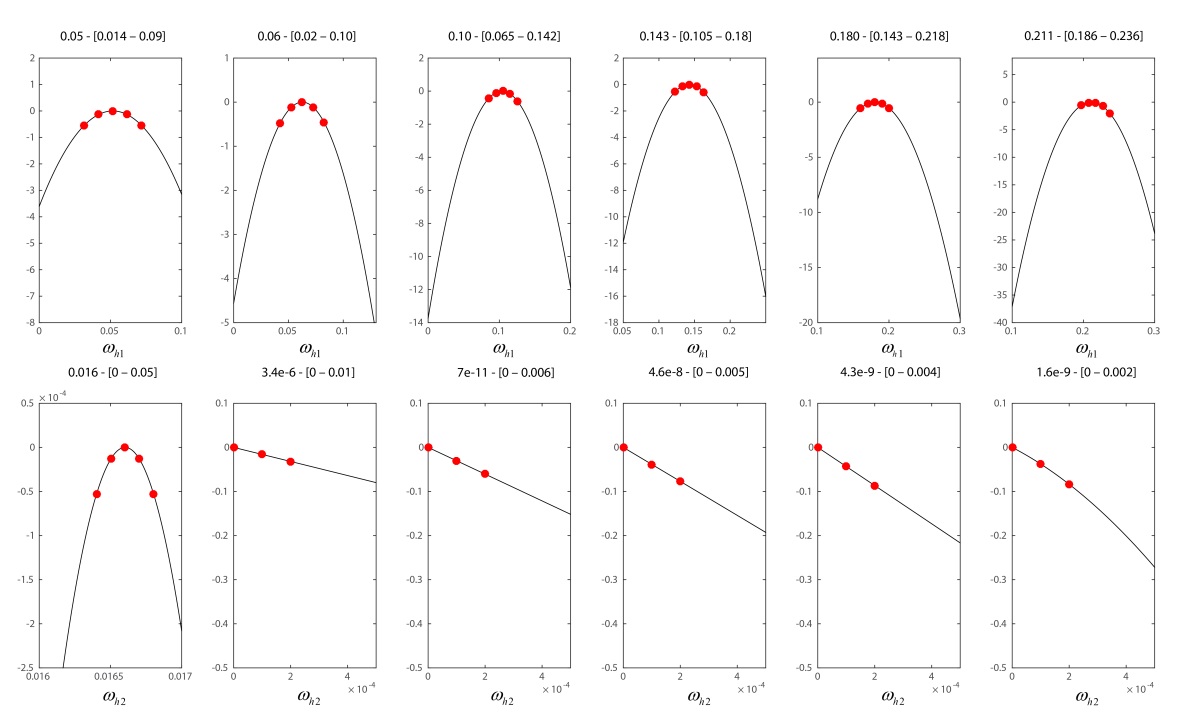
**

Figure S8: Log-likelihood profile of reactivation rates of people in G2 group. (TB) un-infected individuals account for 10% of new hyper-susceptible individuals. The top row panels show log-likelihood profiles of *ω_h1_* – reactivation of hyper-susceptible people with latent TB infection. The bottom row panels show log-likelihood profiles of *ω_h2_* – reactivation of hyper-susceptible with history of active TB. From the left to the right, the expected survival time of hyper-susceptible individuals varies from 1 year to 6 years.


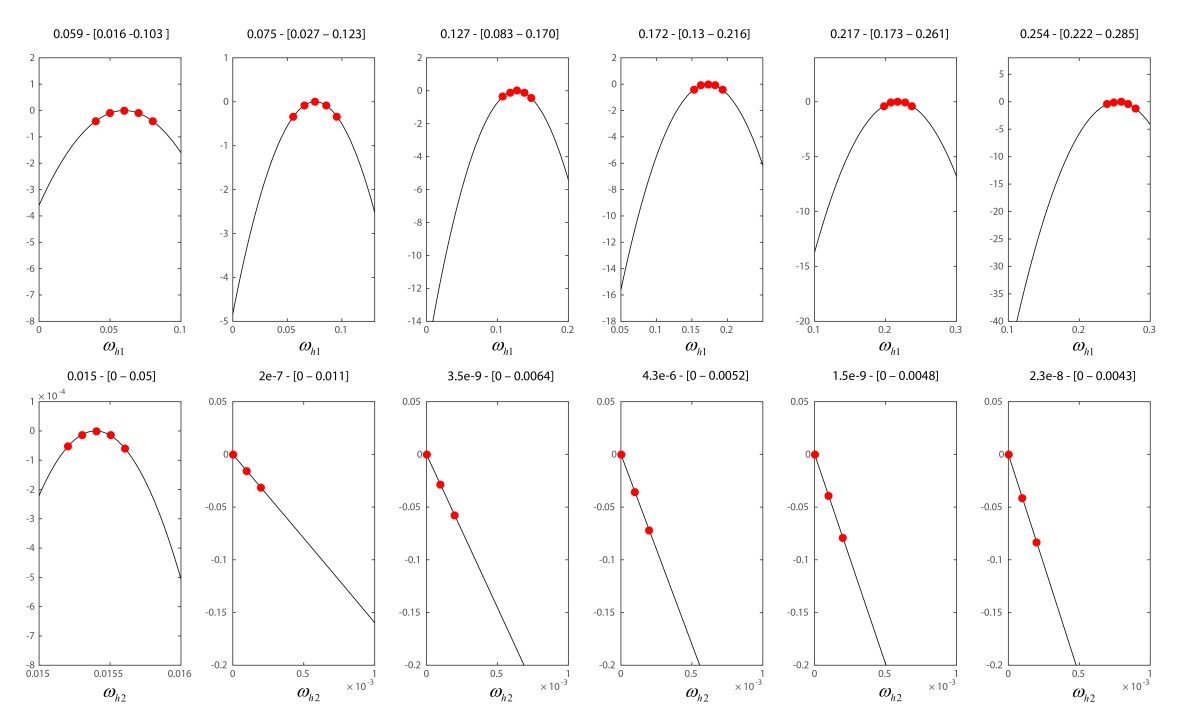


Figure S9: Log-likelihood profile of reactivation rates of people in G2 group. (TB) un-infected individuals account for 20% of new hyper-susceptible individuals. Other settings are similar to Figure S8.


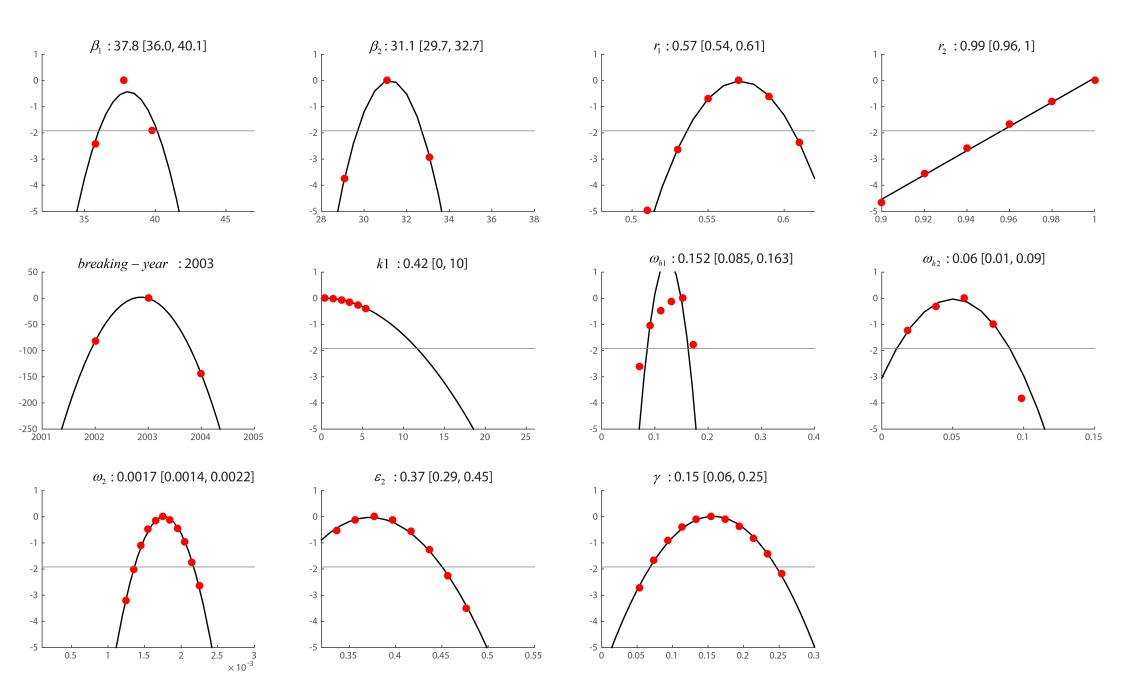


Figure S10: Log-likelihood profile with one-year expected survival time for individuals with hypersusceptibility. The y-axis shows the log-likelihood difference (comparing to MLE). The black line is the quadratic approximation. The area that is above the gray line defines the confidence intervals. Because *breaking − year* takes discrete values, confidence intervals is skipped for this parameter.


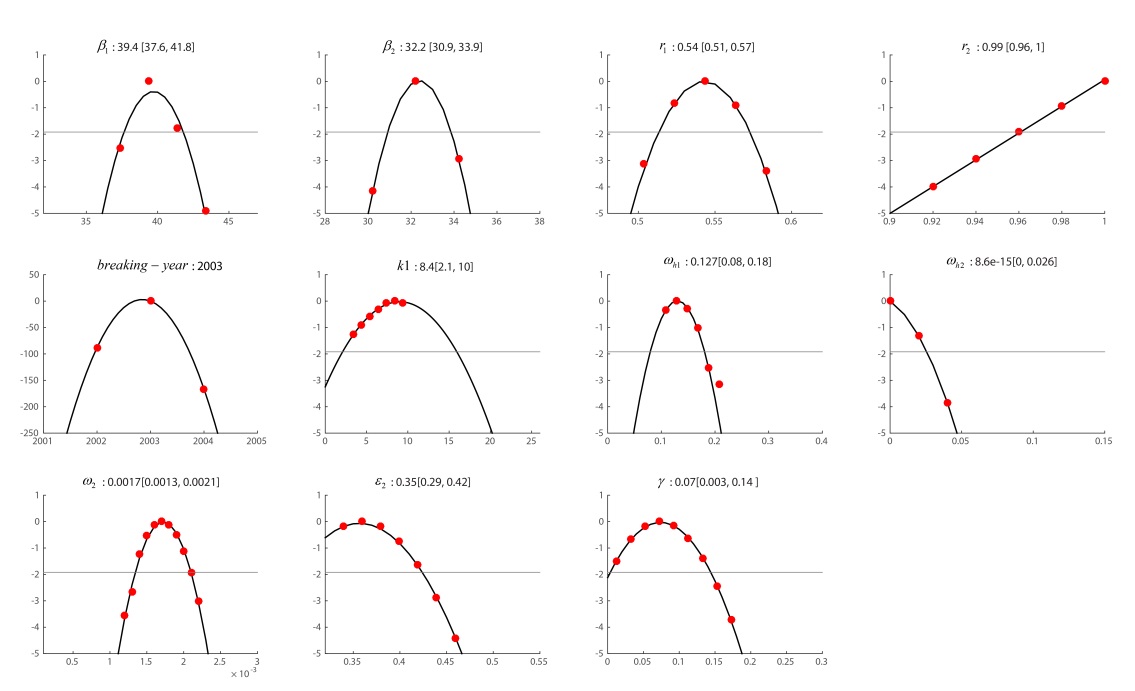


Figure S11: Log-likelihood profile with two-year expected survival time for people with hyper-susceptibility. Other settings are similar to Figure S10.


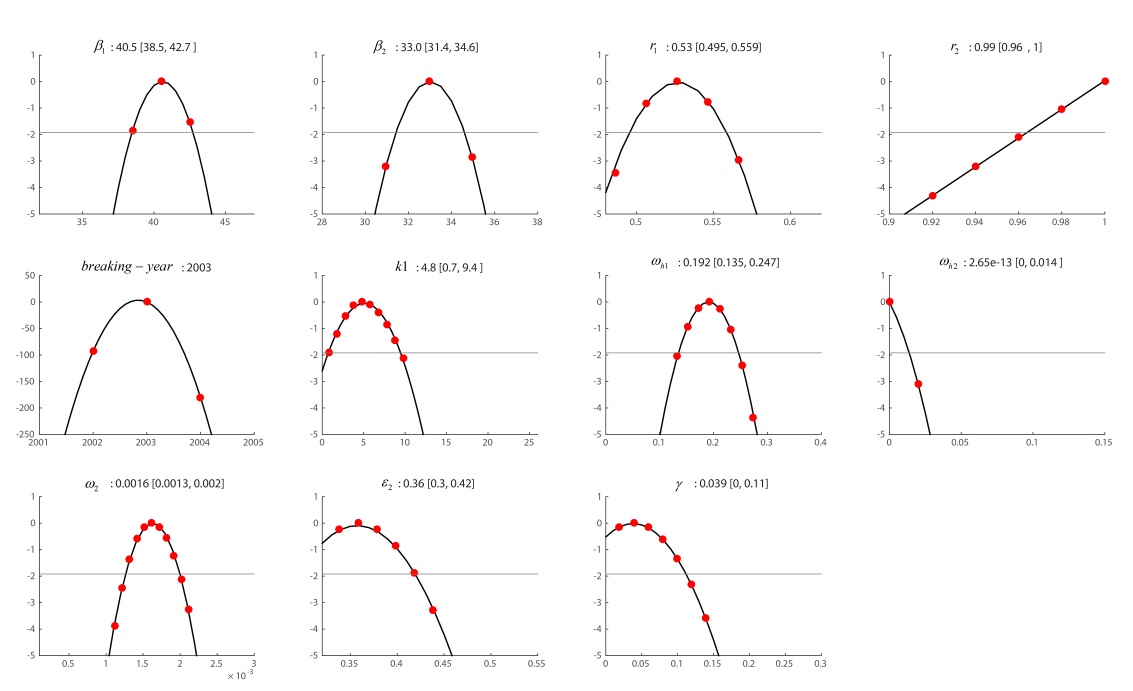


Figure S12: Log-likelihood profile with three-year expected survival time for people with hyper-susceptibility. Other settings are similar to Figure S10.


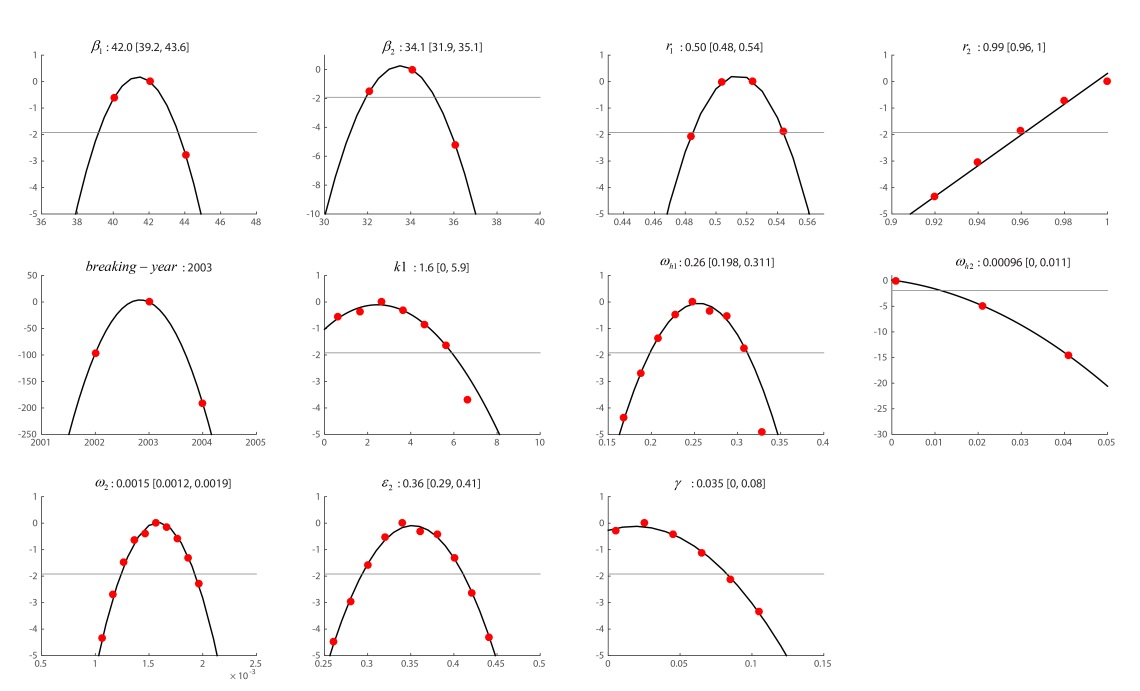


Figure S13: Log-likelihood profile with four-year expected survival time for people with hyper-susceptibility. Other settings are similar to Figure S10.


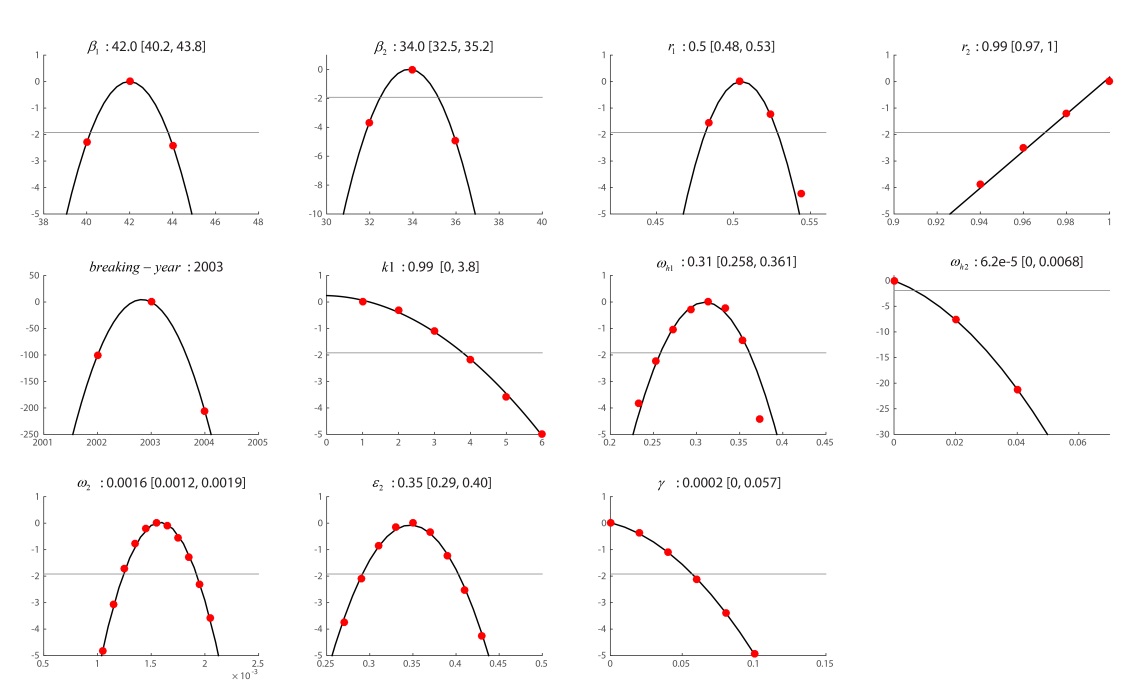


Figure S14: Log-likelihood profile with five-year expected survival time for people with hyper-susceptibility. Other settings are similar to Figure S10.


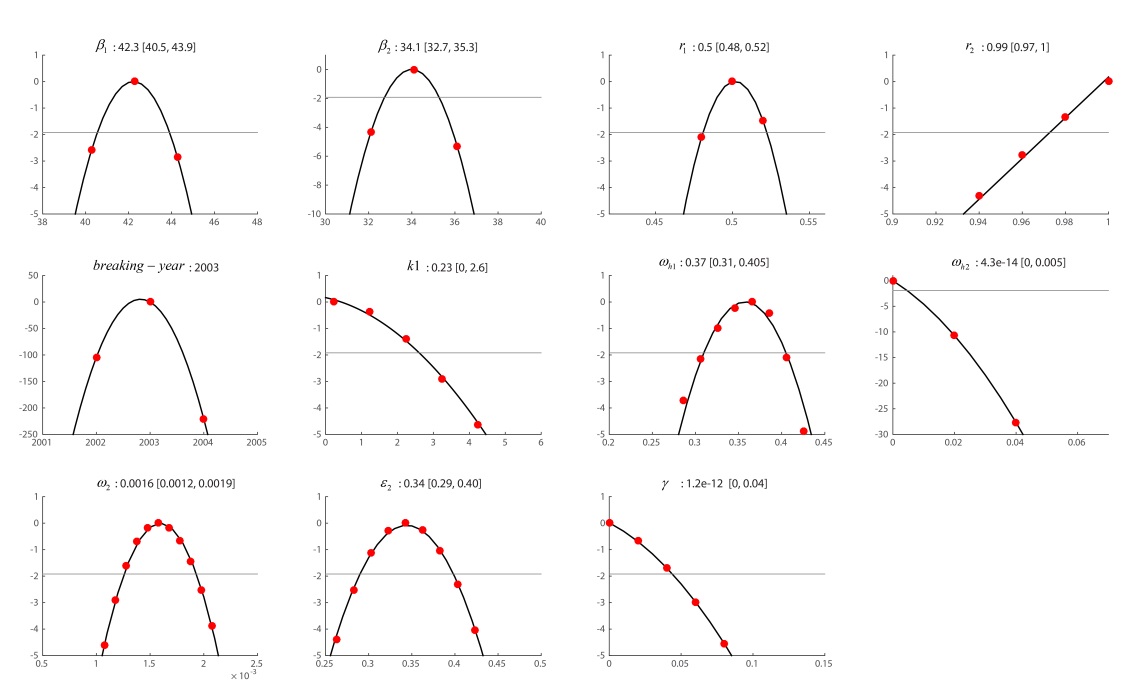


Figure S15: Log-likelihood profile with six-year expected survival time for people with hyper-susceptibility. Other settings are similar to Figure S10.

**8 Tables**

Table S1: Summary of AIC comparison with different epidemiological hypotheses with different values of expected survival time of hyper-susceptible individuals. The value in bold corresponds to the best hypothesis.

| Hypothesis | Assumption | | #optimized parameters | AIC – min(AIC) | | | | | |
| --- | --- | --- | --- | --- | --- | --- | --- | --- | --- |
|  | ***ß(t)*** | ***r_relapsed-tb_(t)*** |  | 1 year | 2 years | 3 years | 4 years | 5 years | 6 years |
| H1 | Constant | Constant | 8 | 1886.96 | 1851.76 | 1857.54 | 1883.04 | 1919.80 | 1958.16 |
| H2 | Time-varying | Constant | 9 | 385.16 | 378.53 | 374.87 | 393.36 | 418.37 | 446.17 |
| H3 | Constant | Time-varying | 10 | 1787.5 | 1791.01 | 1793.16 | 1788.03 | 1790.35 | 1796.15 |
| H4 | Time-varying | Time-varying | 11 | **0.0** | **0.0** | **0.0** | **0.0** | **0.0** | **0.0** |

Table S2: MLE and 95% CIs of parameters in H4. Because *breaking-year* takes discrete values, the confidence interval of this parameter is skipped. The expected survival time of hyper-susceptible individuals varies across [1 year, 6 years]. The non-monotonic trend of *k_1_* is believed to come from the difference in shape of AIDS dynamics when the expected survival time (*est*) varies.

| **Prms** | **Expected Survival Time of Hyper-susceptible Individual (*est*)** | | | | | | | | | | | |
| --- | --- | --- | --- | --- | --- | --- | --- | --- | --- | --- | --- | --- |
|  | **1 year** | | **2 years** | | **3 years** | | **4 years** | | **5 years** | | **6 years** | |
|  | **MLE** | **95% CI** | **MLE** | **95% CI** | **MLE** | **95% CI** | **MLE** | **95% CI** | **MLE** | **95% CI** | **MLE** | **95% CI** |
| *ß_1_* | *37.8* | *[36 – 40.1]* | *39.4* | *[37.6 – 41.8]* | *40.5* | *[38.5 – 42.7]* | *42.0* | *[39.2 – 43.6]* | *42.0* | *[40.2 – 43.8]* | *42.3* | *[40.5 – 43.9]* |
| *ß_2_* | *31.1* | *[29.7 – 32.7]* | *32.2* | *[30.9 – 33.9]* | *33.0* | *[31.4 – 34.6]* | *34.1* | *[31.9 – 35.1]* | *34.0* | *[32.5-35.2]* | *34.1* | *[32.7 – 35.3]* |
| *ε_2_* | *0.37* | *[0.29 – 0.45]* | *0.35* | *[0.29 – 0.42]* | *0.36* | *[0.3 – 0.42]* | *0.36* | *[0.29 – 0.41]* | *0.35* | *[0.29 – 0.4]* | *0.34* | *[0.29 – 0.4]* |
| *ω_2_* | *0.0017* | *[0.0014 – 0.0022]* | *0.0017* | *[0.0013 – 0.0021]* | *0.0016* | *[0.0013 – 0.002]* | *0.0015* | *[0.0012 – 0.0019]* | *0.0016* | *[0.0012 – 0.0019]* | *0.0016* | *[0.0012 – 0.0019]* |
| *k_1_* | *0.42* | *[0 - 10]* | *8.4* | *[2.1 – 10]* | *4.8* | *[0.7 – 9.4]* | *1.6* | *[0 – 5.9]* | *0.99* | *[0 – 3.8]* | *0.23* | *[0 – 2.6]* |
| *ω_h1_* | *0.152* | *[0.09 – 0.163]* | *0.127* | *[0.08 – 0.18]* | *0.192* | *[0.135 – 0.247]* | *0.26* | *[0.2 - 0.31]* | *0.31* | *[0.25 – 0.36]* | *0.37* | *[0.31 – 0.4]* |
| *ω_h2_* | *0.06* | *[0.01 – 0.09]* | *8.6e-15* | *[0 – 0.026]* | *2.6e-13* | *[0 – 0.014]* | *0.00096* | *[0 – 0.011]* | *6.2e-05* | *[0 – 0.0068]* | *4.3e-14* | *[0 – 0.005]* |
| *γ* | *0.15* | *[0.06 – 0.25]* | *0.07* | *[0.003 – 0.14]* | *0.039* | *[0 – 0.11]* | *0.035* | *[0 – 0.08]* | *2.7e-4* | *[0 – 0.057]* | *1.12e-12* | *[0 – 0.04]* |
| *r_1_* | *0.57* | *[0.54 – 0.61]* | *0.54* | *[0.51 – 0.57]* | *0.53* | *[0.49 – 0.56]* | *0.5* | *[0.48 – 0.54]* | *0.5* | *[0.48 – 0.53]* | *0.5* | *[0.48 – 0.52]* |
| *r_2_* | *0.99* | *[0.96 - 1]* | *0.99* | *[0.96 - 1]* | *0.99* | *[0.96 - 1]* | *0.99* | *[0.96 - 1]* | *0.99* | *[0.97 - 1]* | *0.99* | *[0.97 - 1]* |
| *breaking-year* | *2003* | *NA* | *2003* | *NA* | *2003* | *NA* | *2003* | *NA* | *2003* | *NA* | *2003* | *NA* |

Table S3: MLE summary. The force of TB infection of G2 group is 1.5 times higher than G1 group. Only 95%CIs of *ω_h1_* and *ω_h2_* are shown. Other 95%CIs are skipped due to high computation.

| **Prms** | **Expected Survival Time of Hyper-susceptible Individual (*est*)** | | | | | | | | | | | | **Percentage of new hyper-susceptible people are uninfected with TB** |
| --- | --- | --- | --- | --- | --- | --- | --- | --- | --- | --- | --- | --- | --- |
|  | **1 year** | | **2 years** | | **3 years** | | **4 years** | | **5 years** | | **6 years** | |  |
|  | **MLE** | **95% CI** | **MLE** | **95% CI** | **MLE** | **95% CI** | **MLE** | **95% CI** | **MLE** | **95% CI** | **MLE** | **95% CI** |  |
| *ß_1_* | *38.2* |  | *38.9* |  | *40.5* |  | *41.4* |  | *42.1* |  | *42.6* |  | *20%* |
| *ß_2_* | *31.3* |  | *31.7* |  | *32.8* |  | *33.2* |  | *33.6* |  | *33.8* |  |  |
| *ε_2_* | *0.35* |  | *0.35* |  | *0.35* |  | *0.35* |  | *0.33* |  | *0.33* |  |  |
| *ω_2_* | *0.0018* |  | *0.0017* |  | *0.0016* |  | *0.0016* |  | *0.0016* |  | *0.0017* |  |  |
| *k_1_* | *7.1* |  | *7.43* |  | *4.2* |  | *2.2* |  | *0.87* |  | *0.03* |  |  |
| *ω_h1_* | *0.059* | *[0.016 -0.103 ]* | *0.075* | *[0.027 – 0.123]* | *0.127* | *[0.083 – 0.170]* | *0.172* | *[0.13 – 0.216]* | *0.217* | *[0.173 – 0.261]* | *0.254* | *[0.222 – 0.285]* |  |
| *ω_h2_* | *0.015* | *[0 – 0.05]* | *2e-7* | *[0 – 0.011]* | *3.5e-9* | *[0 – 0.0064]* | *4.3e-6* | *[0 – 0.0052]* | *1.5e-9* | *[0 – 0.0048]* | *2.3e-8* | *[0 – 0.0043]* |  |
| *γ* | *0.06* |  | *0.03* |  | *5e-12* |  | *3e-13* |  | *1e-13* |  | *2.4e-8* |  |  |
| *r_1_* | *0.56* |  | *0.55* |  | *0.53* |  | *0.52* |  | *0.5* |  | *0.5* |  |  |
| *r_2_* | *1* |  | *1* |  | *1* |  | *1* |  | *1* |  | *1* |  |  |
| *ß_1_* | *38.03* |  | *38.8* |  | *40.3* |  | *41.2* |  | *42* |  | *42.6* |  | *10%* |
| *ß_2_* | *31.05* |  | *31.6* |  | *32.5* |  | *33.0* |  | *33.4* |  | *33.7* |  |  |
| *ε_2_* | *0.35* |  | *0.35* |  | *0.35* |  | *0.34* |  | *0.33* |  | *0.32* |  |  |
| *ω_2_* | *0.0019* |  | *0.0018* |  | *0.0016* |  | *0.0016* |  | *0.0016* |  | *0.0017* |  |  |
| *k_1_* | *6.8* |  | *7.7* |  | *4.6* |  | *2.5* |  | *1.07* |  | *3e-6* |  |  |
| *ω_h1_* | *0.05* | *[0.014 – 0.09]* | *0.06* | *[0.02 – 0.10]* | *0.10* | *[0.065 – 0.142]* | *0.143* | *[0.105 – 0.18]* | *0.180* | *[0.143 – 0.218]* | *0.211* | *[0.186 – 0.236]* |  |
| *ω_h2_* | *0.016* | *[0 – 0.05]* | *3.4e-6* | *[0 – 0.01]* | *7e-11* | *[0 – 0.006]* | *4.6e-8* | *[0 – 0.005]* | *4.3e-9* | *[0 – 0.004]* | *1.6e-9* | *[0 – 0.002]* |  |
| *γ* | *0.07* |  | *0.03* |  | *2e-11* |  | *3e-13* |  | *5e-17* |  | *2e-10* |  |  |
| *r_1_* | *0.57* |  | *0.56* |  | *0.53* |  | *0.52* |  | *0.5* |  | *0.5* |  |  |
| *r_2_* | *1* |  | *1* |  | *1* |  | *1* |  | *1* |  | *1* |  |  |
|  |  |  |  |  |  |  |  |  |  |  |  |  |  |

**9 Reference**

1. Vietnam Authority of HIV/ AIDS Control. An Annual Update on The HIV Epidemic in Vietnam. 2014;

2. Vietnam Ministry of Health. Annual Report of HIV Epidemic - 2015. 2015;

3. Oxlade O, Murray M. Tuberculosis and poverty: why are the poor at greater risk in India? PLoS One [Internet]. 2012;7:e47533. Available from: http://www.ncbi.nlm.nih.gov/pubmed/23185241

4. Gillies P, Tolley K, Wolstenholme J. Is AIDS a disease of poverty? AIDS Care [Internet]. 1996;8:351–64. Available from: https://www.tandfonline.com/doi/full/10.1080/09540129750125325

5. Muniyandi M, Ramachandran R. Socioeconomic inequalities of tuberculosis in India. Expert Opin. Pharmacother. [Internet]. 2008;9:1623–8. Available from: http://www.tandfonline.com/doi/full/10.1517/14656566.9.10.1623

6. Grange J, Zumla A. Tuberculosis and the poverty-disease cycle. J. R. Soc. Med. [Internet]. 1999;92:105–7. Available from: http://www.ncbi.nlm.nih.gov/pubmed/1297096
